# CTLA-4 Blockade, during HIV Virus-Like Particles Immunization, Alters HIV-Specific B-Cell Responses

**DOI:** 10.3390/vaccines8020284

**Published:** 2020-06-06

**Authors:** Phoebe E. Lewis, Ethan C. Poteet, Dongliang Liu, Changyi Chen, Celia C. LaBranche, Sherry A. Stanfield-Oakley, David C. Montefiori, Guido Ferrari, Qizhi Yao

**Affiliations:** 1 Michael E. DeBakey Department of Surgery, Baylor College of Medicine, Houston, TX 77030, USA; phoebeleonalewis@yahoo.com (P.E.L.); ethan.poteet@gmail.com (E.C.P.); Dongliang.Liu@bcm.edu (D.L.); jchen@bcm.edu (C.C.); 2 Interdepartmental Program in Translational Biology and Molecular Medicine, Baylor College of Medicine, Houston, TX 77030, USA; 3 Duke Human Vaccine Institute, Departments of Medicine, Immunology, Surgery, and Molecular Genetics and Microbiology, Duke University School of Medicine, Durham, NC 27708, USA; celia.labranche@duke.edu (C.C.L.); sherry.oakley@duke.edu (S.A.S.-O.); david.montefiori@duke.edu (D.C.M.); gflmp@duke.edu (G.F.); 4 Center for Translational Research on Inflammatory Diseases (CTRID), Michael E. DeBakey VA Medical Center, Houston, TX 77030, USA

**Keywords:** CTLA-4, HIV, vaccine, VLP, B-cell, Tfh, immunotherapy, antibody

## Abstract

Studies have shown that blockade of CTLA-4 promoted the expansion of germinal center B-cells in viral infection or immunization with model antigens. Few studies have evaluated the immunological consequences of CTLA-4 blockade during immunization against relevant vaccine candidates. Here, we investigated the effects of CTLA-4 blockade on HIV virus-like particles (VLPs) vaccination in a C57BL/6J mouse model. We found that CTLA-4 blockade during HIV VLP immunization resulted in increased CD4+ T-cell activation, promoted the expansion of HIV envelope (Env)-specific follicular helper T cell (Tfh) cells, and significantly increased HIV Gag- and Env-specific IgG with higher avidity and antibody-dependent cellular cytotoxicity (ADCC) capabilities. Furthermore, after only a single immunization, CTLA-4 blockade accelerated T-cell dependent IgG class switching and the induction of significantly high serum levels of the B-cell survival factor, A proliferation-inducing ligand (APRIL). Although no significant increase in neutralizing antibodies was observed, increased levels of class-switched Env- and Gag-specific IgG are indicative of increased polyclonal B-cell activation, which demonstrated the ability to mediate and enhance ADCC in this study. Altogether, our findings show that CTLA-4 blockade can increase the levels of HIV antigen-specific B-cell and antigen-specific Tfh cell activity and impact humoral immune responses when combined with a clinically relevant HIV VLP-based vaccine.

## 1. Introduction

HIV/AIDS remains an incurable disease that currently infects 37 million people worldwide, with over one million people dying from HIV-related complications each year [1], yet a safe and effective vaccine is still not available. Various vaccine strategies have been evaluated in HIV vaccine clinical efficacy trials, but only one study, RV144, showed modest protection with an efficacy rate of 31.2% [2,3,4,5,6,7]. Correlates of risks and protections from these clinical trials provide a framework to design new vaccine strategies against HIV, and these strategies could possibly benefit from incorporating CTLA-4 blockade during immunization [8,9,10,11].

CTLA-4 is an immune checkpoint molecule highly expressed on regulatory T-cells, specifically the T follicular regulatory T-cells (Tfrs) present in the germinal centers [12,13]. Recent studies have demonstrated that CTLA-4 played a critical role in regulating these germinal center (GC) responses [12,13,14,15,16]. The GC reaction is responsible for the production of class switched antibodies by GC B-cells undergoing the processes of somatic hypermutation and class switch recombination. These processes are mediated through survival and costimulatory signals provided by follicular helper T-cell (Tfh) cells and are regulated by Tfrs [12,13,14,15,16,17]. Studies have shown that deletion or blockade of CTLA-4 using anti-CTLA-4 antibodies resulted in the expansion of Tfh cells, GC B-cells, and increased serum IgG [12,13,14,16,18]. Studies evaluating CTLA-4 deletion or blockade during immunization with ovalbumin-based model antigens have also shown an increase in antigen specific antibody responses [16,18]. These studies provide an indication that blocking CTLA-4 during immunization can show utility in vaccine strategies against challenging and clinically relevant vaccine candidates such as HIV.

A protective vaccine strategy against HIV must target the appropriate epitopes and must induce class switching to achieve the appropriate type of antibody response. The C57BL/6J mouse model produces the following IgG subtypes: IgG3, IgG1, IgG2b, and IgG2c. Each subtype has different functional capacities regarding their ability to fix complement and bind to Fc receptors present on effector cells, which play critical roles in protective antibody functions, such as antibody-dependent cellular cytotoxicity (ADCC). ADCC has been the only significant correlate of protection identified in clinical efficacy trials evaluating candidate HIV vaccines [4,8,19,20]. This is an important mechanism of protection in both HIV and cancer, and antibodies capable of mediating this function, such as IgG2c in C57BL/6J mice, can be elicited through vaccination [19,21,22].

The cytokines IL-10 and IL-21, as well as the BAFF (B-cell activating factor of the TNF family) system molecules, are important for B-cell responses. A proliferation-inducing ligand (APRIL) is a part of the BAFF system and can bind to the receptors BCMA (B-cell maturation antigen) or TACI (transmembrane activator and calcium-modulating cyclophilin ligand interactor), which are expressed on B-cells. APRIL has been shown to play a role in class switching and can increase transcription of the enzyme that is critical for this process, AID [23]. APRIL’s receptor, TACI, has also been shown to affect the expansion of Tfh cells [24,25]. Another important cytokine for B-cell responses is IL-10. IL-10 can be produced by a variety of cell types, including B-cells and Tfrs [17,26]. Tfrs express high levels of CTLA-4 that are involved in the regulation of the GC reaction. Recent work has shown that Tfrs can secrete IL-10 which supports the GC reaction [17]. Other studies have demonstrated that APRIL can induce the production of IL-10 from activated B-cells [26]. The hallmark cytokine of Tfh cells is IL-21, which functions as a survival signal to B-cells undergoing multiple rounds of somatic hypermutation.

In this study, we used noninfectious HIV virus-like particles (VLPs) composed of Gag and envelope (Env) which retain the structure of HIV virions without the genetic material required for infection and replication [27,28]. Our VLPs are capable of inducing both cell-mediated and antibody-mediated immune responses that can be amplified or modified through the addition of immune-modulatory molecules such as CD40L, TLR-agonists, or hemagglutinin [27,28,29,30,31,32,33,34,35,36]. This study investigated the effects of CTLA-4 blockade, using a CTLA-4 blocking antibody, on HIV VLP immunization. We evaluated how CTLA-4 blockade, during immunization with a clinically relevant vaccine, affects HIV-specific B-cell responses by analyzing HIV antigen-specific antibody responses over time, and evaluating the expression levels of key cytokines involved in the regulation of the antibody response. Furthermore, we analyzed how CTLA-4 blockade affects T-cell activation and how this blockade can affect the expansion of HIV antigen-specific Tfh cells. Finally, we evaluated how CTLA-4 blockade impacts the key protective properties of vaccine-induced immune responses, by evaluating neutralizing antibodies, antibody avidity and ADCC functions, and establishing T-cell and B-cell memory.

## 2. Materials and Methods:

### 2.1. Cells and Virus-Like Particle (VLP) Production

VLPs used in this study were derived from XC-34 cells (a generous gift from Dr. Spearman at Emory University, Atlanta, GA, USA) engineered to express inducible HIV Gag-IIIB and HIV Envelope-BaL. The VLPs were produced as described previously [29,31,37]. Briefly, VLPs were induced by adding 2 μg/mL of doxycycline to the XC-34 cells. After 6 days, media containing the VLPs was harvested, centrifuged to remove cellular debris, and then filtered through a 0.45 μm filter. VLPs were isolated through high-speed ultracentrifugation at 140,000× *g* for 2 h and resuspended in PBS containing Ca^2+^ Mg^2+^. Properties of HIV VLP were characterized using Western blot, as previously described [27,29].

### 2.2. VLP Envelope (Env) Conformation Analysis

To determine the conformation of Env expressed on the surface of VLPs, VLP-producing XC-34 cells were resuspended in FACS buffer and stained with the broadly neutralizing antibodies (bnAbs) VRC01 (NIH AIDS reagent cat #12033, Germantown, MD, USA), PGT-145 (NIH AIDS reagent cat #12703, Germantown, MD, USA), PGT-121 (NIH AIDS reagent #12343, Germantown, MD, USA), or N6 (NIH AIDS reagent #12968, Germantown, MD, USA) at a concentration of 2 μg/mL for 1 h at room temperature, followed by staining with anti-human IgG AF488 (A-11013 ThermoFisher Scientific, Rokford, IL, USA) at a concentration of 1:1000 for 30 min. then, binding of bnAbs to XC-34 cells was analyzed on an LSR-II, and Flow Jo was used for data analysis.

### 2.3. C57BL/6J Mice Immunization and Specimen Collection

C57BL/6J mice (Jackson Laboratory, Bar Harbor, ME, USA) at 8–12 weeks of age were used in two separate study cohorts (*n* = 15 per cohort). Mice in each cohort were assigned to one of the following three immunization groups (*n* = 5 per group): PBS, VLP, and VLP + anti-CTLA-4 Ab (VLP + CTLA-4 blockade). In Cohort 1, mice were immunized two times intramuscularly (i.m.) with 200 μg of VLPs into the quadriceps at days 0 (prime) and 14 (boost 1). The VLP + anti-CTLA-4 Ab received 200 μg of anti-CTLA-4 Ab (Bio X Cell UC10-4F10-11, West Lebanon, NH, USA) intraperitoneal (i.p.) 1 day before each VLP immunization and 2 additional 100 μg (i.p.) doses 3 days and 6 days after each VLP immunization. For Cohort 1, there are a total of 2 VLP immunizations (prime boost) with the VLP + anti-CTLA-4 blockade group also receiving a total 6 anti-CTLA-4 Ab i.p injections. Cohort 1 was sacrificed 10 days after the second VLP immunization (boost 1). In Cohort 2, we used a similar vaccination regimen as in Cohort 1 but with a third VLP boost (boost 2) on day 28. Cohort 2 was sacrificed 7 days after the third VLP immunization (boost 2), and there was a total of 3 i.m. VLP immunizations (prime, boost 1 and boost 2) and 9 i.p. injections of anti-CTLA-4 Ab. For both cohorts, blood was drawn through submandibular bleeding, 1 day before each immunization. A graphical format for the immunization protocol for both cohorts is shown in Figure S1.

At sacrifice, spleens, lymph nodes, and bone marrow were harvested, and serum was isolated from blood collected through cardiac puncture. Spleens and lymph nodes were processed into single cell suspensions and analyzed by flow cytometry as detailed below. All mice were maintained under specific pathogen-free conditions in the animal facilities of Baylor College of Medicine and in accordance with the animal protocol approved by the Institutional Animal Care and Use Committee (IACUC). The animal protocol AN-3894 was approved on 5/12/2017.

### 2.4. AID-Cre Mice Immunization

To analyze vaccination-induced memory B-cells by our different groups of immunization regimen, we used activation-induced cytidine deaminase (AID)-Cre mice (kindly provided by Drs. Claude-Agnès Reynaud and Jean-Claude Weill, Université Paris Descarte, Paris, French). Rosa mT/mG reporter mice (#007676 Jackson Labs Stock, Bar Harbor, ME, USA) were crossed with Tamoxifen inducible AID-Cre mice to create double transgenic AID-Cre mice which cleave dTomato and express GFP when tamoxifen was present and AID was activated [38]. AID-Cre mice were immunized using a “prime-boost-boost” strategy as described for Cohort 2. Tamoxifen (10 mg) (TCI, Portland, OR, USA) was administered through oral gavage (250 μL at 40 mg/mL) on days 6, 11, 15, and 29. Mice were sacrificed 10 weeks after receiving the third and final VLP immunization (boost 2) and spleens, lymph nodes, and bone marrow were harvested.

### 2.5. Flow Cytometry Analysis

Splenocytes or lymph node mononuclear cells (LMNCs) were resuspended in FACS staining buffer (PBS, 2% BSA, 5 mM EDTA, and 0.03% NaN3), stained, and then fixed in Cytofix/Cytoperm (BD Bioscience, San Jose, CA). T-cell activation panel consisted of CD3 AF700 (500-A2, BD Pharminogen, San Diego, CA), CD4 PE (GK1.5, BD Pharminogen, San Diego, CA, USA), CD8 PE-CF594 (53-6.7 BD Horizon, San Jose, CA, USA), and CD69 APC (H1.2F3, Biolegend, San Diego, CA, USA). Germinal center B-cell panel consisted of CD3 AF700 (500-A2, BD Pharminogen, San Diego, CA, USA), B220 PE-Cy7 (RA3-6B2 BD Pharminogen, San Diego, CA, USA), CD95 PE-CF594 (Jo2, BD Pharminogen, San Diego, CA, USA), and GL7 BV421 (GL7, BD Horizon, San Jose, CA, USA). Memory T-cell Panel consisted of CD3 AF700 (500-A2, BD Pharminogen, San Diego, CA, USA), CD4 BV650 (GK1.5, BD Horizon, San Jose, CA, USA), CD8 BV786 (53-6.7, BD Horizon, San Jose, CA, USA), CD62L APC (MEL-14 Biolegend, San Diego, CA, USA), and CD44 FITC (IM7, Biolegend, San Diego, CA, USA). Memory B-cell panel consisted of CD3 AF700 (500-A2, BD Pharminogen, San Diego, CA, USA), B220 PE-Cy7 (RA3-6B2, BD Pharminogen, San Diego, CA, USA), and CD73 AF650 (TY/23, BD Pharminogen, San Diego, CA, USA).

### 2.6. Antigen-Specific Follicular T-Cell Assay

LMNCs from Cohort 2 were harvested at sacrifice and single cell suspensions were generated. LMNCs were stimulated with consensus B HIV Gag peptides (NIH AIDS reagent cat #8117, Germantown, MD, USA) or consensus B HIV Env peptides (NIH AIDS reagent cat #9480, Germantown, MD, USA) or left unstimulated for 6 h, and then surface stained with CD3 AF700 (500-A2, BD Pharminogen, San Diego, CA), CD4 BV650 (GK1.5, BD Horizon, San Jose, CA, USA), and PD-1 AF488 (J43, eBioscience, San Diego, CA, USA). The, cells were fixed with Cytofix/Cytoperm (BD Bioscience, San Jose, CA, USA) and intracellular stained for IL-21 efluor-660 (mhalx21, eBioscience, San Diego, CA, USA). Cells were analyzed on BD LSR-Fortessa (BD Biosciences, Franklin Lakes, NJ, USA). FlowJo software (Ashland, O, USA) was used for data analysis. The percent change of peptide stimulated from non-stimulated was calculated to determine peptide-specific IL-21+Tfh cells.

### 2.7. Serum IgG ELISA

Micro-vinyl plates (96-well, Corning Costar, Corning, NY, USA) were coated with 0.5 µg/mL of BaL gp120 Env recombinant protein (NIH AIDS reagent #4961, Germantown, MD, USA) or Gag IIIB recombinant protein (NIH AIDS reagent #3276, Germantown, MD, USA) in sodium bicarbonate buffer and incubated overnight at 4 °C. Plates were washed three times with 0.05% PBST and blocked with 5% BSA in PBS. Mouse serum was diluted at 1:500 in PBS and incubated overnight, then washed 5 times with 0.05% PBST before goat anti-mouse IgG HRP secondary antibody (Cell signaling #7076S, Danvers, MA, USA), diluted 1:20,000 in 5% BSA in PBS, was added to each well for 2 h. After washing 6 times with 0.05% PBST, the 1-Step™ Turbo TMB-ELISA (Thermo Scientific #34022, Rockford, IL, USA) reagent was added and incubated for 20 min at room temperature. Sulfuric acid (2N) was used to stop the reaction. Plates were analyzed on a spectrophotometer and optical density was measured at 450 nm.

### 2.8. Quantitative IgG Subtype ELISA

Micro-vinyl plates (96-well, Corning Costar, Corning, NY, USA) were coated with a standard curve of purified IgG1 (BD Pharminogen #554121, San Diego, CA, USA), IgG2b (eBioscience eBMG2b #14732-82, San Diego, CA, UAS), IgG2c (GeneTex #GTX35043, Irvine, CA, USA), or IgG3 (eBioscience #14-4742-82, San Diego, CA, USA) protein while remaining wells were coated with 0.5 μg/mL BaL gp120 recombinant protein (NIH AIDS reagent #4961, Germantown, MD, USA) in a sodium bicarbonate buffer and incubated overnight. Plates were washed with 0.05% PBST 3 times and blocked with 5% BSA in PBS. Mouse serum was diluted in PBS at 1:500 for IgG2b and IgG3 and 1:1000 for IgG1 and IgG2c, and incubated overnight at 4 °C. The following day, plates were washed 5 times with 0.05% PBST and one of the following secondary goat anti-mouse HRP antibodies were added for 2 h: anti-Mouse IgG1 (Southern Biotech 1070-05, Birmingham, AL, USA), anti-Mouse IgG2b (Southern Biotech 1090-05, Birmingham, AL, USA), anti-Mouse IgG2c (Southern Biotech 1079-05, Birmingham, AL, USA), or anti-Mouse IgG3 (Southern Biotech 1100-05, Birmingham, AL, USA). After washing 6 times with 0.05% PBST, 1-Step™ Turbo TMB-ELISA (Thermo Scientific #34022, Rockford, IL, USA) reagent was added and incubated for 20 min at room temperature. Sulfuric acid was used to stop the reaction. Plates were analyzed on a spectrophotometer at 450 nm.

### 2.9. Western Blot for A Proliferation-Inducing Ligand (APRIL)

Serum from Cohort 2 was diluted 1:100 in RIPA and Laemmli buffer and loaded in a 15% gel for SDS-PAGE. Each 15-well gel was loaded with a protein ladder and serum from 4 PBS mice, 5 VLP immunized mice, and 5 VLP + anti-CTLA-4 Ab immunized mice. After an overnight transfer, membranes were incubated with APRIL antibody (1:1000 dilution) (eBioscience TALL-2, TRDL-1a, San Diego, CA, USA) overnight, followed by a 2 h incubation with goat anti-mouse IgG-HRP (Cell signaling #7076S, Danvers, MA, USA).

### 2.10. Serum Cytokine Analysis

Immunization is used to refer to the injection of VLPs. The serum collected after each immunization is referred to as post-prime, post-boost 1, or post-boost 2 serum. Post-prime serum was collected 13 days after the first VLP immunization; post-boost 1 serum was collected 11–13 days after the second VLP immunization; and post-boost 2 serum was collected 7 days after the third VLP immunization. Serum from these various time points was diluted 1:10 and analyzed using Legendplex mouse Th1/Th2 cytokine bead array (Biolegend #740005, San Diego, CA, USA) according to manufacturer’s instruction and analyzed through flow cytometry on an LSR Fortessa. This cytokine bead array, based on the same principles used in sandwich immunoassays, quantifies the concentration of a given cytokine in the sample with a standard curve of known concentrations.

### 2.11. HIV Neutralization Analysis

Serum samples collected at sacrifice from Cohorts 1 and 2 were sent to the Center for AIDS research at Duke University for neutralizing antibody analysis using TZM-bl assay, as described previously [39]. Briefly, serum was analyzed against strains HIV-MN.3, HIV-Bal.26, and the nonspecific negative control, SVA-MLV. Values reported were the serum dilution at which relative luminescence units (RLUs) were reduced 50% as compared with virus control wells (no serum).

### 2.12. Avidity Analysis

Micro-vinyl plates (96-well, Corning Costar, Corning, NY, USA) were coated with 0.5 µg/mL of BaL gp120 Env recombinant protein (NIH AIDS reagent #4961, Germantown, MD, USA) or Gag IIIB recombinant protein (NIH AIDS reagent #3276, Germantown, MD, USA) in sodium bicarbonate buffer and incubated overnight at 4 °C. Plates were washed two times with 0.05% PBST and blocked with 5% BSA in PBS. Duplicate wells containing mouse serum diluted at 1:500 in PBS were incubated overnight. The following day, diluted serum was removed and one of the duplicate wells was treated with 7M urea for 20 min, while the other well was treated with 0.05% PBST. After incubation, wells were washed 4 more times with 0.05% PBST, then incubated with goat anti-mouse IgG HRP secondary antibody (Cell signaling #7076S, Danvers, MA, USA), diluted 1:20,000 in 5% BSA in PBS for 2 h. After washing 6 times with 0.05% PBST, the 1-Step™ Turbo TMB-ELISA (Thermo Scientific #34022, Rockford, IL, USA) reagent was added and incubated for 20 min at room temperature. Sulfuric acid (2N) was used to stop the reaction. Plates were analyzed on a spectrophotometer and optical density was measured at 450 nm. The avidity index was calculated by taking the OD450 value of the urea treated well divided by the untreated well, as previously described [40,41].

### 2.13. Antibody Dependent Cellular Cytotoxicity

The ADCC activity was measured by Dr. Guido Ferrari’s lab at the Center for AIDS research at Duke University utilizing a modified version of previously published ADCC luciferase procedure [42]. Briefly, CEM.NKRCCR5 cells (NIH AIDS Reagent #4376, Germantown, MD, USA) were used as targets for ADCC luciferase assays after infection with subtype B HIV-1 infectious molecular clone (IMC)_BaL_ (GenBank accession AY426110, NIH AIDS Reagent #11414, Germantown, MD, USA) [43]. In order to account for possible Ab responses against human Ag included in the vaccine preparation, SIVmac251-infected cells (NIH AIDS reagent #133, Germantown, MD, USA) were also tested and utilized to determine background against nonspecific Ags. The NK92 cells, transduced to express murine CD16 by the Ferrari’s lab, were used as the effector cells and prepared according to published protocol [44]. An effector to target cell ratio equal to 10:1 was used for ADCC analysis. For each sample, percent specific killing was measured in two wells using six 4-fold dilutions starting at 1:200. Cocultures were incubated for 6 h at 37 °C in 5% CO_2_. The final readout was the reduction of luminescence intensity (RLU) generated by the presence of residual intact target cells that have not been lysed by the effector population in the presence of ADCC-mediating plasma Abs. The percentage of killing was calculated using the following formula:

% specific killing = [(RLU of target and effector well − RLU of test well)/(RLU of target and effector well)] × 100

In this analysis, the RLU of the target plus effector wells represents spontaneous lysis in the absence of any source of Ab and used to calculate the background. The RSV-specific monoclonal antibody palivizumab and a cocktail of HIV-1 monoclonal Abs (A32, 2G12, CH44, and 7B2) were used as negative and positive controls, respectively. A positive response is defined as peak activity greater than or equal to 10% after baseline subtraction and having peak activity greater than or equal to 10% after subtracting the activity against the SIVmac251-infected target cells. ADCC activities are reported either as the maximum percent specific killing observed for each test serum at any dilution or as ADCC titers, defined as the serum dilution that intersects the positive cutoff at ≥10% specific killing.

### 2.14. Statistical Analysis

Data from PBS, VLP, and VLP + anti-CTLA-4 Ab groups are presented as the arithmetic mean ± standard error mean (SEM). Statistical analyses were done using a one-way ANOVA and Tukey’s range test for comparison of parametric data. The Shapiro–Wilk Test was used to determine that data was normally distributed. The Mann–Whitney test was used for comparing two non-parametric means. Unpaired T-test was used to compare means between two groups. Graphpad Prism was used to calculate statistics (Graphpad Software, Inc., La Jolla, CA, USA). A value of *p* < 0.05 was considered significant.

## 3. Results

### 3.1. CTLA-4 Blockade with VLP Vaccination Results in Increased CD4+ T-Cell Activation

**Figure 1:**
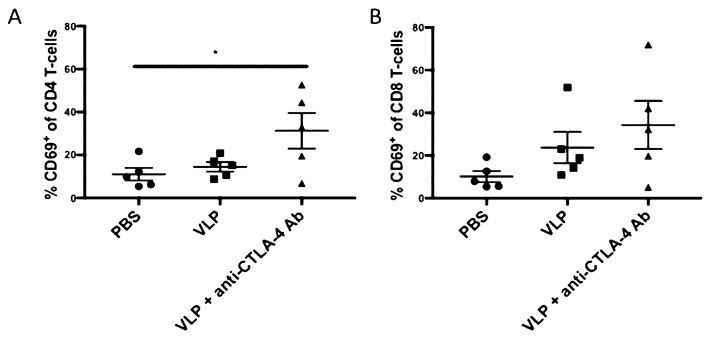
CTLA-4 blockade enhances CD4 T-cell activation. Splenocytes from Cohort 1 were harvested 10 days after receiving their second virus-like particle (VLP) dose, and 4 days after receiving their final injection of anti-CTLA-4 Ab. CD3+ T-cells were pre-gated and CD4 and CD8 discrimination gating was performed (Figure S2A). Cumulative results from all animals are shown for (**A**) CD4+ CD69+ T-cells and (**B**) CD8+ CD69+ T-cells. Statistical significance was determined using a one-way ANOVA and Tukey post-hoc analysis for multiple comparisons. A line on top of two groups indicates statistical differences between these two groups. * *p* < 0.05.

To determine the effects that CTLA-4 blockade had in our anti-CTLA-4 Ab + VLP vaccination on T-cell subsets we harvested splenocytes from mice in Cohort 1 at 10 days after they received their second and final VLP immunization (post-boost 1) and 4 days after receiving their final anti-CTLA-4 Ab injection. The expression of the activation marker CD69, on both CD4+ and CD8^+^ T-cells was determined by flow cytometry and the gating strategy is shown in Figure S2. We found that CD69 was significantly upregulated in mice receiving VLPs + anti-CTLA-4 Ab as compared with PBS controls (31% vs. 11%, *p* = 0.0436) on the CD4+ T-cell subset (Figure 1A). The differences between PBS and VLPs (11% vs. 14.5%, *p* = 0.8884) or VLPs and VLPs + CTLA4-blockade (14.5% vs. 31%, *p* = 0.0976) did not reach statistical significance. In the CD8+ T-cell subset, we observed a similar trend in CD69 upregulation, but it did not reach statistical significance (Figure 1B). We also performed an experiment using CTLA-4 blockade only as a baseline control in C57BL/6J mice and checked the CD4+ and CD8+ T-cell activation as compared with PBS as a negative control; and we found no statistical differences between these two groups (Figure S3). This demonstrates that CTLA-4 blockade in combination with HIV VLP immunization can increase activation of CD4+ T-cells.

### 3.2. CTLA-4 Blockade Induces Increased HIV Env-Specific Tfh Cells

**Figure 2:**
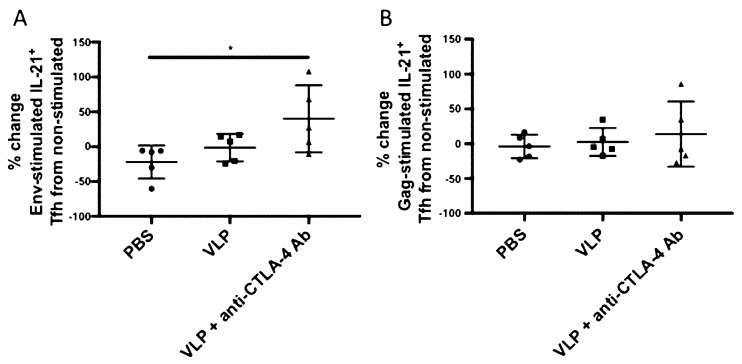
CTLA-4 blockade enhances Env-specific IL-21+ Tfh cells. Lymph nodes were harvested from Cohort 2 at sacrifice 7 days after they received their third and final VLP immunization (post-boost 2). Single cell suspensions were generated, and cells were incubated with gag- or Env-peptide (2 μg/mL) for 6 h, then surface stained for CD3 AF700, CD4 BV650, and PD-1 AF488 (gating strategy shown in Figure S4). After stimulation, cells were fixed, permeabilized, and stained for intracellular IL-21 efluor 660. Cells were analyzed on LSR-Fortessa and FlowJo software was used for data analysis. HIV peptide-specificity is expressed as IL-21 percent change from non-stimulated in the CD4+ PD1Hi Tfh subset. (**A**) Env-specific IL-21+ Tfh cells. (**B**) Gag-specific IL-21+ Tfh cells. Statistical significance was determined using a one-way ANOVA and Tukey post-hoc analysis for multiple comparisons. A line on top of two groups indicates statistical differences between these two groups. * *p* < 0.05.

To determine whether our immunization strategy utilizing CTLA-4 blockade could increase antigen-specific Tfh cells in LMNCs, we measured changes in HIV antigen-specific IL-21+ Tfh induction among the three immunization groups. LMNCs from Cohort 2 were either left unstimulated or stimulated with Env or Gag peptides for 6 h, then stained for surface Tfh markers and intracellular IL-21. Antigen specificity was calculated by taking the percent change of IL-21+ Tfh cells in the antigen-stimulated condition to non-stimulated condition for each mouse ((antigen stimulated–non-stimulated)/non-stimulated) (gating strategy is shown in Figure S4). We found that CTLA-4 blockade significantly increased Env-specific IL-21+ CD4+ PD-1^Hi^ T-cells as compared with PBS (~40% change from non-stimulated, *p* = 0.0459) (Figure 2A). No significant increased Gag-specific Tfh cells were observed (Figure 2B). This demonstrates that CTLA-4 blockade, when combined with HIV VLP immunization, can increase Tfh cells in an antigen-specific manner.

### 3.3. VLP Immunization Independent of CTLA-4 Blockade Promotes Increased Germinal Center B-Cells

**Figure 3:**
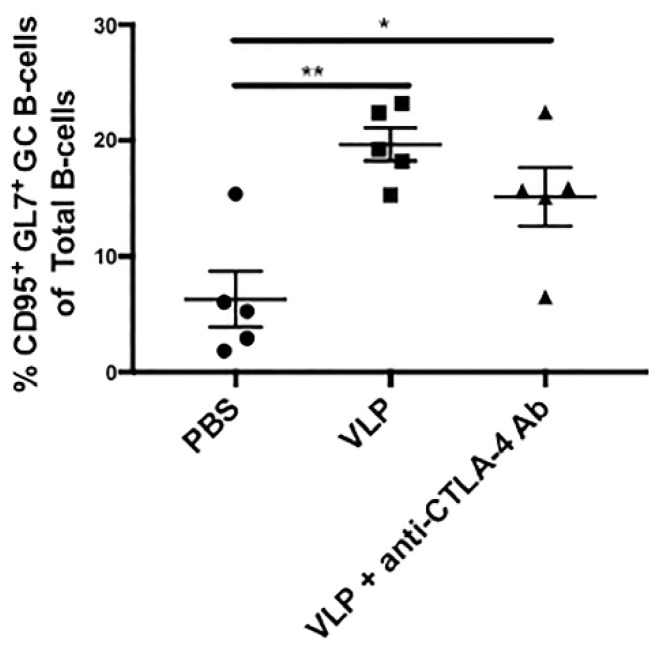
VLP Immunization increases germinal center B-cells. LMNCs were isolated from lymph nodes harvested from Cohort 2 mice, 7 days after the third and final VLP immunization (post-boost 2). LMNCs were stained for CD3 AF700, B220 PE-Cy7, CD95 PE-CF594, and BV421 GL7. B-cells were gated as CD3-B220+, and germinal center B-cells were identified as CD95+ GL7+ (Figure S5). CD95+ GL7+ germinal center B-cells are plotted as the percentage of total CD3- B220+ B-cells for each animal. Statistical significance was determined using a one-way ANOVA and Tukey post-hoc analysis for multiple comparisons. A line on top of two groups indicates statistical differences between these two groups. * *p* < 0.05 and ** *p* < 0.01.

Tfh cells secrete IL-21 as a hallmark cytokine critical for B-cells undergoing germinal center reactions [45]. To determine whether the increase in Env-specific Tfh cells was accompanied by an increase in germinal center B-cells, we analyzed CD3-B220+ B-cells from LMNCs of mice from each immunization group in Cohort 2 for the germinal center markers CD95 and GL7. The gating strategy is shown in Figure S5. As shown in Figure 3, we found that VLP immunization in the presence or absence of CTLA-4 blockade resulted in increased CD3- B220+ CD95+ GL7^+^ germinal center B-cells as compared with PBS (PBS, ~6.3% vs. VLP, ~19.7% *p* = 0.0026 and PBS, ~6.3% vs. VLP + anti-CTLA-4 Ab, ~15.1% *p* = 0.0350). There were no statistically significant differences between the VLPs alone, and VLPs + anti-CTLA-4 Ab groups (*p* = 0.3422). This demonstrates that HIV VLP immunization alone, independent of CTLA-4 blockade, can promote the expansion of germinal center B-cells.

### 3.4. CTLA-4 Blockade Results in Increased HIV-Specific Antibody Responses, Polyclonal B-Cell Activation, and Class Switch Recombination Favoring IgG1 and IgG2c

**Figure 4:**
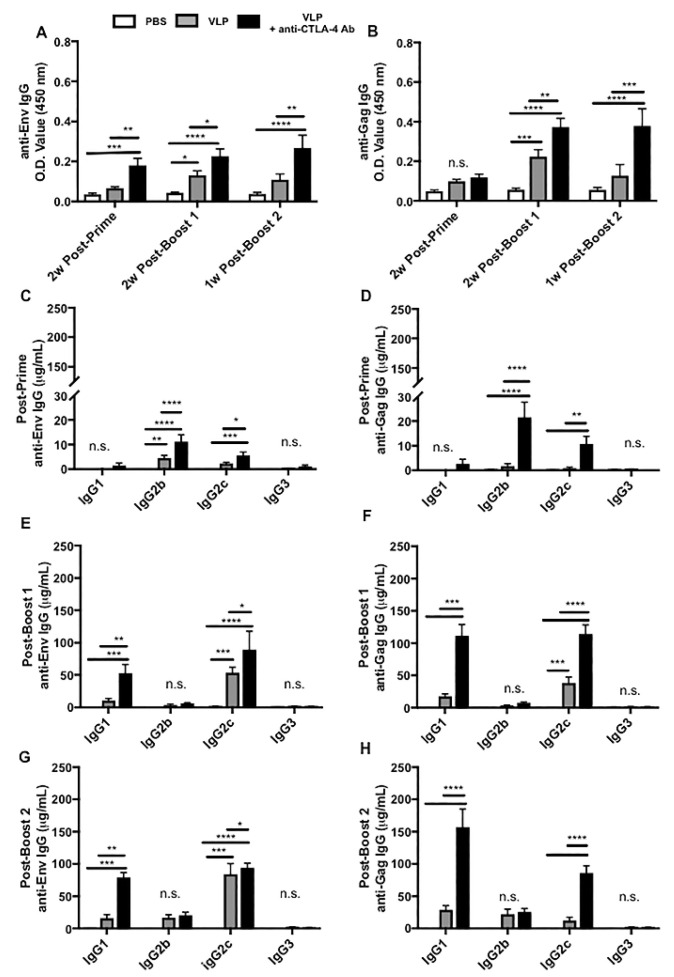
CTLA-4 blockade amplifies serum HIV Gag- and Env-specific antibody responses. Post-prime serum was collected 13 days after the first VLP immunization; Post-boost 1 serum was collected 11–13 days after the second VLP immunization. Post-prime and Post-boost 1 time points analyzed serum from 10 mice per immunization group from Cohorts 1 and cohorts 2. The Post-boost 2 time point analyzed serum from the 15 mice (5 per group) in Cohort 2 and was collected 7 days after the third VLP immunization. (**A**) Total Env-specific IgG and (**B**) total Gag-specific IgG were analyzed through ELISA. Quantitative ELISAs with standard curves for IgG1, IgG2b, IgG2c, and IgG3 were performed on all serum samples from Cohorts 1 and 2 to detect HIV Env- and Gag-specific IgG subtypes. Shown at each of the following time points: (**C**) post-prime, (**D**) Env post-prime Gag, (**E**) post-boost 1 Env, (**F**) post-boost 1 Gag, (**G**) post-boost 2 Env, and (**H**) post-boost 2 Gag IgG subtypes. The results above are the cumulative results of two independent experiments. P-values were determined by one-way ANOVA and Tukey post-hoc analysis for multiple comparisons. A line on top of two groups indicates statistical differences between these two groups. **** *p* < 0.0001, *** *p* < 0.001, ** *p* < 0.01, * *p* < 0.05, and n.s. *p* > 0.05.

To determine whether CTLA-4 blockade could result in enhanced antibody production, sera from Cohorts 1 and 2 were analyzed for HIV Env- and Gag-specific IgG through ELISA. We found that two VLP immunizations (post-boost 1) were required to induce significant Env- or Gag-specific IgG in the VLP only immunization group, but in the presence of CTLA-4 blockade, significant levels of Env-specific IgG were accomplished with only a single VLP immunization (post-prime), providing evidence that CTLA-4 blockade can accelerate the humoral immune response during VLP immunization (Figure 4A). Significant levels of Gag-specific IgG were not achieved for any immunization group until the second VLP immunization (post-boost 1) (Figure 4B). Here, we show that CTLA-4 blockade during VLP immunization consistently and significantly increased the levels of HIV-specific IgG production as compared with VLP immunization alone and the PBS group.

To better understand the functional qualities of the HIV-specific IgG induced in the presence or absence of CTLA-4 blockade we performed quantitative ELISAs on serum from Cohorts 1 and 2 for each of the four subtypes of mouse IgG that could be induced during class switching. For the VLP immunization alone group, we found modest but significant levels of Env-specific IgG2b (~4.5 μg/mL) detectable after VLP priming and diminished upon the induction of the Th1-dependent IgG2c subclass after boosting (Figure 4C,E,G and Table S1). After the second VLP immunization (post-boost 1), we saw significant levels of Env-specific IgG2c and Gag-specific IgG2c (~53.5 μg/mL and 38.3 μg/mL, respectively) as compared with the PBS group (Figure 4E,F and Table S1). After the third VLP immunization (post-boost 2), we observed that it had significantly higher levels of Env-specific IgG2c (~84 μg/mL) as compared with the PBS group (Figure 4G and Table S1). The addition of CTLA-4 blockade to VLP immunization not only enhances the magnitude of the HIV-specific IgG induction, but it also accelerates the induction of T-cell dependent IgG subtypes. In mice that received VLP immunization in the presence of CTLA-4 blockade we found that after a single VLP prime (post-prime), there were significantly higher levels of three different subtypes of IgG, specific to both Env and Gag (Env-specific IgG2b ~11.3 μg/mL, Gag-specific IgG2b ~27 μg/mL, and Env-specific IgG2c ~5.6 μg/mL) (Figure 4C,D and Tables S1 and S2). Similar to what was observed with VLP immunization alone, we saw that the IgG2b levels diminished after successive VLP immunizations (post-boost 1 and post-boost 2), with the induction of significantly higher levels of Env- and Gag-specific IgG1 and IgG2c (Env-specific IgG1 ~52.7 μg/mL, Gag-specific IgG1 ~115.5 μg/mL, Env-specific IgG2c ~89.2 μg/mL, and Gag-specific IgG2c ~114.4 μg/mL) (Figure 4E,F and Tables S1 and S2). These same subtypes persisted and increased after a third (post-boost 2) VLP immunization + anti-CTLA-4 Ab (Env-specific IgG1 ~79.23 μg/mL, Gag-specific IgG1 ~157.3 μg/mL, Env-specific IgG2c ~94 μg/mL, and Gag-specific IgG2c ~85.9 μg/mL) (Figure 4G–H and Tables S1 and S2). These results reveal that in addition to increasing the levels of HIV-specific IgG subtypes, CTLA-4 blockade also results in increased polyclonal plasma-cell activation indicated by the significant increases in both Th2-associated IgG1 and Th1-associated IgG2c towards both HIV Env and Gag as compared with VLP immunization alone which induces a predominantly Th1-associated IgG2c response towards Env with a limited Gag IgG response.

### 3.5. CTLA-4 Blockade Accelerates the Induction of APRIL in the Sera

**Figure 5:**
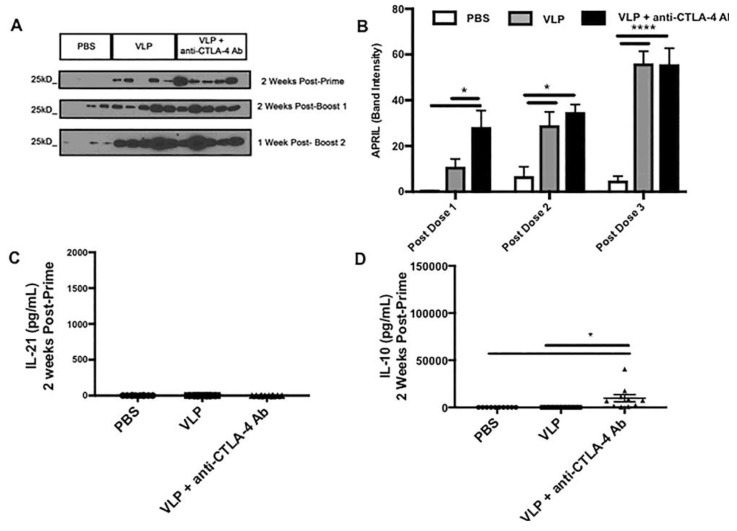

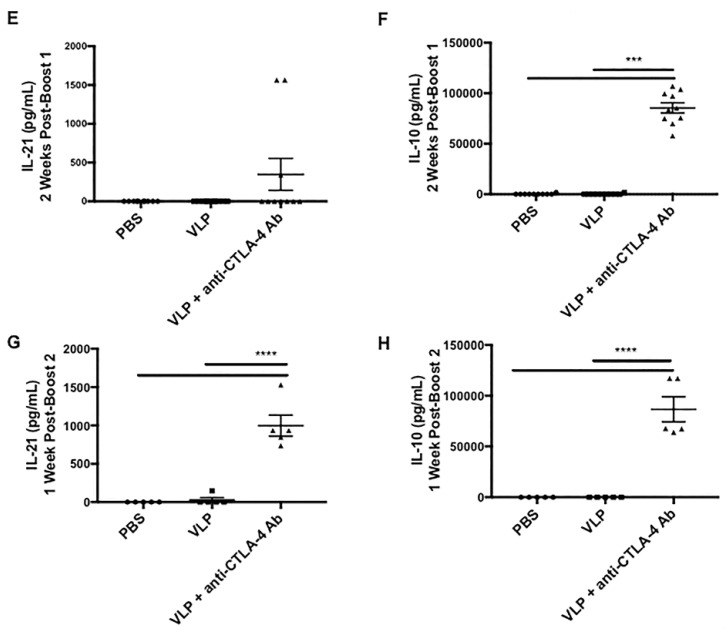
CTLA-4 blockade accelerates the induction of the plasma cell survival factor, A proliferation-inducing ligand (APRIL), and serum cytokines IL-10 and IL-21. Serum from Cohort 2 was diluted 1:100 in RIPA and Laemmli buffer and loaded for SDS-PAGE. Each 15-well gel was loaded with ladder and serum from 4 PBS mice, 5 VLP immunized mice, and 5 immunized VLP + anti-CTLA-4 Ab mice. (**A**) Western blot of APRIL at the indicated time points; (**B**) Densitometric analysis of APRIL expression levels. Serum samples from Cohorts 1 and 2 were analyzed for cytokines using Legendplex™ bead-based immunoassay. Serum samples (*n* = 30) were analyzed in duplicate and cytokine concentrations were calculated from a standard curve; (**C**) IL-21 post-prime serum levels; (**D**) IL-10 post-prime serum levels; (**E**) IL-21 post-boost 1 serum levels; (**F**) IL-10 post-boost 1 serum levels; (**G**) IL-21 post-boost 2 serum levels; (**H**) IL-10 post-boost 2 serum levels. Significance was determined by a one-way ANOVA and Tukey post-hoc analysis for multiple comparisons. A line on top of two groups indicates statistical differences between these two groups. **** *p* < 0.0001, *** *p* < 0.001, and * *p* < 0.05.

To understand how CTLA-4 blockade affects other factors involved in inducing a potent-plasma cell response, we analyzed serum from Cohort 2 immunized mice for the B-cell survival cytokine APRIL. We found that CTLA-4 blockade accelerated the induction of significantly high levels of APRIL after a single VLP immunization. APRIL increased with successive VLP immunizations, and serum levels of APRIL became comparable to the VLP alone group after two immunizations (Figure 5A). Figure 5B is the serum APRIL protein quantitation plot based on the Western blot shown in Figure 5A (Figure S6). This indicates that CTLA-4 blockade accelerates the induction of APRIL after priming, and this could be involved in the early T-cell dependent class switching to IgG2c and induction of various subclasses of HIV-specific IgG we observed (Figure 4C).

### 3.6. CTLA-4 Blockade during VLP Immunization Increases Serum Levels of IL-10 and IL-21

To determine CTLA-4 blockade effects on cytokines in the serum, we performed multiplex Th1/Th2 cytokine assays at three time points after each immunization. With the addition of CTLA-4 blockade, we observed significantly elevated IL-10 after a single VLP prime (Figure 5D), and the levels increased with successive VLP boosts (Figure 5F,H). Without CTLA-4 blockade, there was no IL-10 induction, as observed in the VLP only immunization group. The cytokines IL-10 and IL-21 have been shown to play critical roles in the germinal center response [17,45]. In addition, we found that similar to what we observed in the lymph nodes, serum IL-21 level was elevated significantly after the third VLP immunization in the presence of CTLA-4 blockade (Figure 5G). As IL-21 is the hallmark cytokine of Tfh cells, this could indicate enhanced peripheral Tfh activity [45,46].

### 3.7. CTLA-4 Blockade Does Not Improve HIV Neutralizing Antibody Responses with VLP Immunization

Serum from Cohorts 1 and 2 collected at the time of sacrifice was analyzed for neutralizing antibody activity against HIV-BaL.26, HIV-MN, and the control virus MLV, using the TZM-bl cell-based assay [39]. We observed no significant neutralizing activity against the virus HIV-BaL.26; however, there was a trend towards neutralization against HIV-MN in the VLP group (*p* = 0.0848, Mann–Whitney test PBS-MN vs. VLP-MN) that was not seen in the VLP + anti-CTLA-4 Ab group. We also observed no significant differences in titers between mice receiving two VLP immunizations as compared with those receiving three VLP immunizations. Of note, C57BL/6J mice are known to exhibit spontaneous neutralizing activity, which could explain the activity in the PBS group (Table 1).

### 3.8. CTLA-4 Blockade Induces High Avidity HIV-Specific Antibodies

**Figure 6:**
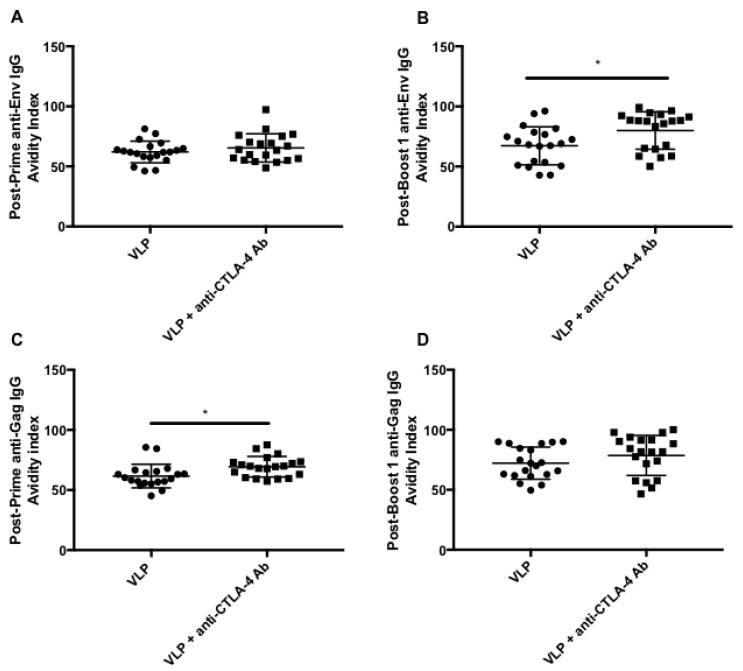
CTLA-4 blockade increases the avidity of HIV-specific antibody responses. The avidity index of serum from Cohorts 1 and 2 (*n* = 30) was analyzed through a urea-based avidity assay. (**A**) Post-prime anti-Env IgG avidity index; (**B**) Post-boost 1 anti-Env IgG avidity index; (**C**) Post-prime anti-Gag IgG avidity index; (**D**) Post-boost 1 anti-Gag IgG avidity. Significance was determined by an unpaired T-test. * *p* < 0.05. Data is representative of two independent experiments.

Affinity maturation is a critical component of the germinal center reaction and results in the induction of high affinity antibodies [47,48]. An avidity analysis, utilizing chaotropic agents such as urea can be used to interrogate the affinity of the antibody response, by measuring how tightly antibodies bind to a given antigen in the presence of a disassociating chaotropic agent [40,41]. The avidity index describes the percentage of reactivity remaining after treatment with urea [49]. We performed avidity analysis on post-prime and post-boost 1 serum from mice immunized with VLPs or VLPs + anti-CTLA-4 Ab. The PBS serum was not analyzed because no significant Env- or Gag-specific IgG antibodies were observed in ELISA analysis (Figure 4 and Tables S1 and S2). We found similar avidity indexes after priming with the VLP group demonstrating a post-prime anti-Env IgG avidity index of ~62% and VLP + anti-CTLA-4 Ab group demonstrating a post-prime anti-Env IgG avidity index of ~65%, *p* = 0.3124 (Figure 6A). However, post-boost-1 serum demonstrated a significant increase in the Env-specific IgG avidity index with the VLP group demonstrating an avidity index of ~67% and the VLP + anti-CTLA-4 Ab group demonstrating an avidity index of ~80% *p* = 0.0149 (Figure 6B). We also observed a significant increase in the Gag-specific avidity index in the post-prime serum, with the VLP group having a post-prime anti-Gag-specific IgG avidity index of ~62% while the VLP + anti-CTLA-4 Ab group demonstrated a post-prime anti-Gag IgG avidity index of ~69% *p* = 0.0110 (Figure 6C). There was no significant difference in the post-boost 1 anti-Gag IgG avidity indexes with the VLP group having an avidity index of ~72% and the VLP + anti-CTLA-4 Ab group having a post-boost 1 anti-Gag IgG avidity index of ~79% *p* = 0.1866 (Figure 6D). This demonstrates that CTLA-4 blockade during VLP immunization can increase antibody affinity maturation resulting in an antibody response with a higher avidity.

### 3.9. CTLA-4 Blockade Increases Antibody-Dependent Cellular Cytotoxicity (ADCC) Activity

**Figure 7:**
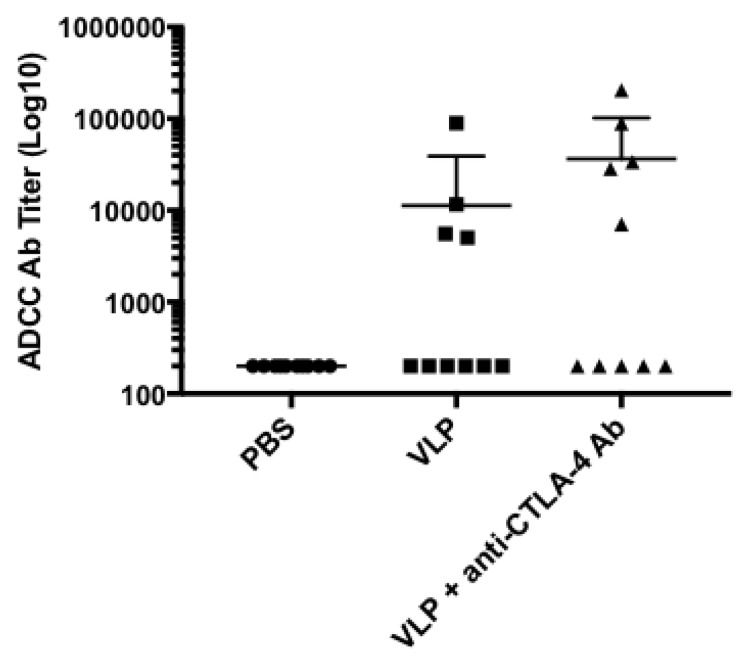
CTLA-4 blockade increases antibody-dependent cellular cytotoxicity (ADCC) activity. CEM.NKRCCR5 cells were infected with HIVBaL or SIVMac251 infectious molecular clones at an effector to target cell ratio of 10:1 and incubated with serial 4-fold dilutions of mouse sera from each immunization group. ADCC activity was detected by the reduction in luciferase activity. Reported ADCC activity has been subtracted against baseline and the activity observed against the SIVMac251-infected target cells. ADCC activities are reported as ADCC titers, defined as the serum dilution that intersects the positive cutoff at ≥10% specific killing. Y-axis is ADCC Ab titer. Each dot represents ADCC activity detected in each individual mouse.

Utilizing an ADCC assay in which CEM.NKRCCR5 target cells were infected with HIV_BaL_ or SIV_Mac251_ infectious molecular clones presenting Env in its native conformation, we found HIV-1BaL-specific ADCC activity in four out of 10 VLP immunized mice, and in five out of 10 VLP + anti-CTLA-4 Ab immunized mice, while there was no ADCC activity observed in serum from the PBS group (Figure 7). Furthermore, the addition of CTLA-4 blockade resulted in higher ADCC titers, demonstrating its ability to improve ADCC function, when combined with a VLP-based HIV vaccine. We observed significant ADCC activity against target cells expressing SIV_Mac251_ resulting in a high-background subtraction of the ADCC activity observed against HIV_BaL_ (data not shown). Altogether, we demonstrate that the addition of CTLA-4 blockade to VLP immunization results in increased HIV-1_BaL_-specific ADCC titers, which corresponds with the increased levels of Env-specific IgG observed in our ELISA analysis (Figure 4).

### 3.10. VLP Immunization, Independent of CTLA-4 Blockade, Induces Increased AID Activated Memory B-Cells and Memory T-Cells

**Figure 8:**
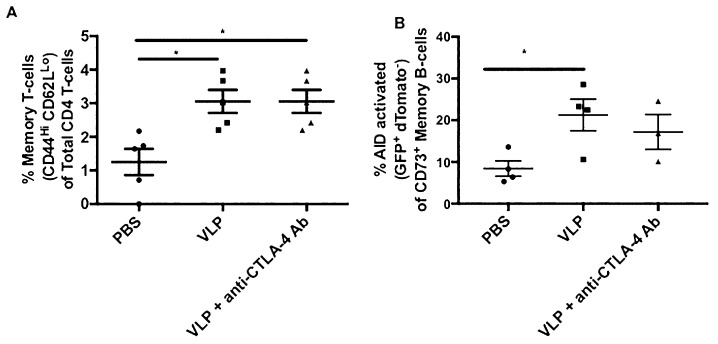
Memory T-cell and B-cell responses are CTLA-4 blockade independent. (**A**) CD4+ CD44Hi CD62LLo memory T-cell analysis. Splenocytes from Cohort 1 were stained with CD3 AF700, CD4 BV650, CD8 BV786, CD62L APC, and CD44 FITC. The percent of CD4+ CD44Hi CD62LLo memory T-cells is plotted for each immunization groups. Gating strategy is shown in Supplementary Figure S8A; (**B**) Percent of AID-activated (GFP+ dTomato-) cells within the CD73+ (memory) B-cell compartment isolated from bone marrow of AID-reporter mice 10 weeks after the third VLP immunization. Bone marrow cells were stained with CD3 AF700, B220 PE-Cy7, and CD73 AF650. Gating strategy for AID-activated memory B-cells is shown in Supplementary Figure S8B. Statistical significance was determined using a one-way ANOVA and Tukey post-hoc analysis for multiple comparisons. A line on top of two groups indicates statistical differences between these two groups. * *p* < 0.05.

Development of immunological memory is important for effective vaccines. We found a significant increase in CD44Hi CD62LLo memory T-cells in mice receiving VLP immunization, both with or without CTLA-4 blockade, that increased from ~1.25% to ~3.06% in the VLP and VLP+ anti-CTLA-4 Ab groups (Figure 8A). We also interrogated B-cell memory using an AID-Cre reporter mouse model that irreversibly labeled AID-activated B-cells as GFP+ dTomato- cells. We observed a significant increase in AID-activated CD73+ memory B-cells in the bone marrow of VLP immunized mice 10 weeks after the last immunization from ~8.4125% in the PBS group to ~21.25% in the VLP group (Figure 8B). We also observed a similar trend in mice receiving VLPs in the presence of CTLA-4 blockade (~17.2%); however, it did not reach statistical significance. Altogether, we find that VLP immunization alone is sufficient to induce memory T-cell and B-cell responses, and CTLA-4 blockade does not improve cellular memory responses.

## 4. Discussion

Recent mechanistic studies have highlighted the importance of CTLA-4 in regulating the germinal center response, which could have major implications in vaccine development [12,13,16,50,51]. Here, we show that CTLA-4 blockade with HIV VLP immunization results in CD4^+^ T-cell activation, the promotion of Env-specific IL-21+Tfh cells, and a significant increase in serum HIV Gag- and Env-specific IgG. Despite consistent and substantially higher levels of HIV Env-specific IgG, we failed to observe any significant levels of HIV neutralizing antibodies in the group receiving VLPs in combination with CTLA-4 blockade at the time points that we analyzed. VLP immunization, in the presence or absence of CTLA-4 blockade, was capable of inducing increased germinal center B-cells, as well as T-cell and B-cell memory. Furthermore, CTLA-4 blockade resulted in increased antigen-specific polyclonal B-cell activation and class switching, indicated by the induction of high levels of various Env- and Gag-specific IgG subtypes with high avidity, and high ADCC activity.

Using the early activation marker, CD69, to measure activation on the CD4+ and CD8+ T-cell subsets after CTLA-4 blockade, we found a significant increase in CD4+ T-cell activation and a trend in CD8+ T-cell activation (Figure 1). CTLA-4 blockade has been shown to increase CD4+ T-cell activation in previous studies. For example, CTLA-4 blockade, particularly when combined with PD-1 blockade, enhanced activation, proliferation, and expansion of LN and PB memory CD4+ and CD8+ T-cells in rhesus macaques infected with SIV [52]. Therefore, we interrogated the CD4+ T-cell subset further to examine the specialized subset of CD4+ T-cells known as Tfh cells [53,54]. A thorough analysis of T-cell activation could be best done with multiple T-cell activation markers, however, we focused this study primarily around the striking increase in antibody responses that we observed. Although CD69 alone could be diluted by proliferation, all the samples were analyzed and stained at the same time, therefore, any dilution due to proliferation would be expected to be seen uniformly. Despite these limitations, we still observed a significant increase in CD69 expression on CD4 T-cells which was not observed on CD8 T-cells in mice splenocytes that were immunized with VLPs + anti-CTLA-4 blockade. This supports data by a report that showed that CTLA-4 was more highly expressed on CD4 T-cells as compared with CD8 T-cells [55].

Several studies have demonstrated that deleting or blocking CTLA-4 could result in the expansion of Tfh cells [12,13,16,56]. These Tfh cells play a critical role in sending survival signals to GC B-cells undergoing somatic hypermutation and class switch recombination, through secretion of their hallmark Tfh cytokine IL-21, as well as through contact-dependent co-stimulation [57]. In a longitudinal study that evaluated a large group of HIV infected individuals from acute infection, a correlation between circulating Tfh cells and the induction of bnAbs was identified [46]. These bnAbs are capable of neutralizing a wide variety of HIV strains in vitro and in vivo through passive transfer and typically exhibit high levels of somatic hypermutation, which is mediated by Tfh cells [58,59,60,61,62,63,64,65]. Our study demonstrates, in the setting of a clinically relevant vaccine candidate that CTLA-4 blockade can result in the expansion of antigen-specific IL-21+Tfh, as well as an increase in circulating IL-21 in the serum, which warrants further investigation into circulating Tfh cells (Figure 2 and Figure 5G). In this study, we classified Tfh as being lymph node mononuclear cells that were IL-21+ CD3+ CD4+ PD-1+, which accepted markers for Tfh cells in the literatures [60,61,62]. We omitted CXCR5 from this panel for fluorophore compatibility reasons. We have shown in previous publications that VLP immunization can increase total Tfh cells, defined as CD3+ CD4+ PD-1+ CXCR5+ cells [36]. In the current study, however, we interrogated antigen-specificity and Tfh functionality by looking at the hallmark Tfh cytokine, IL-21, under various peptide stimulation conditions. We also observed an increase in GC B-cells, although the expansion of this population seemed to be driven by VLP immunization rather than CTLA-4 blockade, as both the VLP and VLP + anti-CTLA-4 blockade groups exhibited increased GC B-cells as compared to PBS (Figure 3).

CTLA-4, highly expressed on Tfrs, is critical for the GC response by regulating the interaction between GC B-cells and Tfh, as well as GC B-cells with Tfrs [66,67,68]. We found a consistent upregulation of IL-10 throughout immunization (Figure 5D,F,H). Various cell types secrete IL-10, including Tfrs [17]. CTLA-4 blockade temporarily inhibits contact-dependent Tfr suppression of GC responses, but contact-independent cytokine secretions are not suppressed. A recent study demonstrated that Tfr-derived IL-10 can activate the GC response to promote the differentiation of plasmablasts and GC B-cells [17]. Studies that investigate the cellular source of increased serum IL-10 with CTLA-4 blockade during vaccination strategies, and whether IL-10 is working in a GC suppressive or an activating manner would be of interest [17,26]. Tfrs are CD4+ T-cell subsets within the germinal centers that both express high levels of PD-1 [57,69,70]. However, we did not perform additional staining or sorting to distinguish the Tfr subset from the Tfh subset because we sought to characterize how the antigen-specific functional activity within the germinal centers and to understand how modulating immune checkpoints on T-cells can influence antibody responses. For this reason, we specifically isolated and stained cells from the lymph nodes, stimulated with Env or Gag peptides, and measured changes in intracellular IL-21 from antigen stimulated to non-stimulated animals. IL-21 is the hallmark Tfh cytokine required for the survival of germinal center B-cells and the production of antibodies. Sage et al. demonstrated that CTLA-4 was more highly expressed on Tfrs as opposed to Tfh cells [13].

The lack of significant Gag-stimulated IL-21 Tfh secretion (Figure 2B) could correspond with the lack of significant anti-gag IgG avidity responses observed in Figure 6D. CTLA-4 blockade + VLP immunization resulted in a subtle, but significant increase in anti-Gag IgG avidity that was observed after priming, but diminished over time as multiple immunizations elicited anti-Gag IgG with a similar avidity to that in mice immunized with VLPs alone. The IL-21 Tfh assay was a cell-based assay that utilized lymph node mononuclear cells harvested at sacrifice, and the cohorts of mice evaluated in this study were sacrificed after receiving multiple immunizations. One explanation, supported by the data in the current study, is that the Gag-specific antibody response has an earlier temporal profile than Env-specific antibody responses. This would explain why cell-based assays performed on lymph node mononuclear cells isolated after multiple immunizations detect Env-specific responses (Figure 2A), but not Gag-specific responses (Figure 2B), while serum collected throughout the immunization process can detect anti-gag IgG responses (Figure 4D,F,H) and highlight significant differences in anti-Gag IgG magnitude, subtype, and avidity (Figure 6C) that occur after a single immunization in mice immunized with VLPs + anti-CTLA-4 antibody.

In addition to the cellular changes we observed, our study demonstrated that CTLA-4 blockade could also increase HIV-specific antibody responses. We interrogated how CTLA-4 blockade affected all four antigen-specific IgG subtypes over time, shedding additional light on potential functional capabilities of these antibodies and how that could contribute immune protection against HIV. We found that CTLA-4 blockade after multiple VLP immunizations induced increased HIV-specific IgG1, in addition to increased levels of HIV-specific IgG2c which VLP immunization alone typically induces (Figure 4 and Tables S1 and S2). This indicates that CTLA-4 blockade can promote both Th2 and Th1 responses against vaccine immunogens. Our study highlights the dynamics of the antibody response and demonstrates how multiple immunizations against a vaccine containing two different antigens, can evolve with successive immunizations. VLP immunization can induce a durable anti-Env IgG response (Figure 4A,C,E,G), which is significantly increased in magnitude, when immunization is performed with CTLA-4 blockade. We observed a more dynamic anti-Gag IgG response in mice immunized with VLPs alone, as well as mice immunized with VLP + anti-CTLA-4 antibody. Rather than a diminished antibody response, we observed a shift in anti-Gag IgG subtypes from IgG2c to IgG1 in both the VLP immunized group and the VLP + anti-CTLA-4 antibody group (Figure 4B,F,H).

CTLA-4 blockade combined with VLP immunization can cause increased polyclonal B-cell activation, as shown by the presence of significantly higher levels of HIV-specific IgG of multiple subclasses directed at both Gag and Env, in contrast to the primarily Env-specific IgG2c response observed in the VLP alone immunization group. Despite CTLA-4 blockade amplifying the magnitude of the antibody response, we failed to observe a significant neutralizing antibody response at the time points that we evaluated (Table 1). The lack of inducing a specific neutralizing antibody response could be attributed to the following two factors specific to our study: the early time points that we interrogated for neutralizing antibodies and the animal model that we used. In our study design, we initially sought to investigate how CTLA-4 blockade influenced both T-cell and B-cell responses, and the peak time to observe T-cell responses is seven to ten days post immunization [71]. Neutralizing antibodies, especially HIV bnAbs, have been known to take years to develop in humans, and months to develop in mice [60,63,64]. In previous studies using the same VLPs utilized in this study, both our group and another group observed neutralizing antibodies in mice, two months after immunization [32,72]. The VLPs used in this immunization study present Env in a conformation recognizable by the bnAbs VRC01, PGT-121, PGT-145, and N6 which demonstrate that the Env present on the surface of the VLPs can display conformational epitopes that are present in the CD4 binding site and V1V2/ V3 regions targeted by bnAbs (Figure S7) [65,73,74,75].

A second factor could have influenced our inability to detect significant neutralizing antibodies. The long heavy-chain complementarity determining region 3 (HCDR3) and its propensity to be polyreactive makes bnAb unique and presents a challenge when using the C57Bl/6J mouse model which is unable to produce antibodies with long HCDR3s [47,76,77]. The HCDR3 plays a key role in influencing the antibody repertoire, which is critical for inducing an antibody response of adequate breadth, capable of recognizing and neutralizing diverse strains of HIV. The process of VDJ recombination determines the length of a B-cell’s HCDR3. Most B-cells have a HCDR3 of around 16 amino acids, while the HCDR3s exhibited by many classes of bnAbs span between 20 and 34 amino acids. The prevalence of naïve B-cells with HCDR3s greater than 24 amino acids is less than 3.5% [77]. Exacerbating the low prevalence of naïve B-cells capable of producing bnAbs with long HCDR3s, is the tendency of B-cells with long HCDR3s to be eliminated through the process of negative selection, as these features are commonly observed in auto-reactive B-cell clones [77,78]. Another important factor concerning the animal model evaluated in this study is that the C57BL/6J mouse model can exhibit nonspecific, spontaneous neutralization, as we observed in the PBS group in this study. High levels of nonspecific spontaneous neutralizing activity in the PBS group made it difficult to delineate between vaccine-induced and spontaneous neutralization. The limited quantities of serum that could be collected in our mouse model also presented limitations on the neutralizing assay. Sera volumes were not adequate enough to heat inactivate the serum, for risk of evaporation; therefore, components of the complement system were also present and could contribute to false positive neutralization activity. Alternative animal models, such as rabbits, transgenic/humanized mouse models, or non-human primates are more suitable animal models to specifically interrogate the induction of bnAbs and whether or not the addition of CTLA-4 blockade can improve neutralizing antibody responses [79,80,81,82,83]. A recent report in rhesus macaques and bnAb immunoglobulin knock-in (KI) mice that express diverse precursors of the CD4 binding site of HIV-1 bnAbs supports our hypothesis by demonstrating that co-administering antibodies targeting both CTLA-4 and PD-1 along with HIV Env vaccines augmented the HIV-1 Env antibody responses, increased germinal center B and Tfh cells, and plasma neutralizing antibodies [84].

In the current study, we observed that the antibody affinity maturation in response to CTLA-4 blockade/VLP immunization is different for Env and Gag in mice. We analyzed anti-Env and anti-Gag-specific IgG avidity in VLP immunized mice +/− anti-CTLA-4 blockade. We also analyzed serum after priming and after boosting to determine whether anti-CTLA-4 blockade could influence anti-Env and anti-Gag affinity maturation. In Figure 6A,B, we show that VLP immunization, in combination with CTLA-4 blockade results in significantly increased anti-Env IgG avidity after two immunizations as compared with VLP immunization alone. Quantitative anti-Env IgG subtype analysis shows that anti-CTLA-4 blockade + VLP serum transitions from a primarily IgG2b and IgG2c response after priming (Figure 4C), to a primarily IgG1 and IgG2c response after boosting (Figure 4E). The increase in magnitude of anti-Env IgG1 and IgG2c observed after boosting (Figure 4E) can explain the increased anti-Env avidity observed in Figure 6B. In Figure 6C,D, we show that VLP immunization, in combination with CTLA-4 blockade results in significantly increased anti-Gag IgG avidity after one immunization (Figure 6C) as compared with VLP immunization alone; however, after two immunizations (Figure 6D), anti-Gag IgG avidity is similar to VLP immunization alone. Quantitative anti-Gag IgG subtype analysis, in Figure 4, shows that the addition of anti-CTLA-4 blockade can cause a significant increase in the magnitude of anti-Gag IgG2b and IgG2c after priming (Figure 4D) that transitions into a primarily anti-Gag IgG1 and IgG2c response after two immunizations (Figure 4F). Anti-Gag avidity after priming (Figure 6C) could correspond with the early induction of anti-Gag IgG2c observed in Figure 4D. After two immunizations, however, the avidity of anti-Gag IgG is also increased in VLP immunized mice and the significance of anti-CTLA-4 blockade is lost, which could correspond with the accompanying increase in anti-Gag IgG2c in mice immunized with VLPs alone (Figure 4F). Overall, we show that anti-CTLA-4 blockade subtly increases both anti-Env and anti-Gag IgG avidity; however, it does so by following a different temporal pattern, which could correspond with IgG class-switching. The production of high affinity antibodies is an indication of successful priming by an antigen and indicates that B-cell clones specific to such antigens have undergone affinity maturation. A recent study found variation in affinity to different antigens over time and differential antibody affinity to different antigens [85]. Since Env and Gag induces differential immune responses, their antibody affinity maturation process should also be different. However, the detailed mechanism of what determines these differences requires further investigation.

Although we did not observe significant levels of neutralizing antibodies, we did find that CTLA-4 blockade in combination with VLP immunization could increase the avidity of antigen-specific IgG (Figure 6). Higher avidity is achieved during the germinal center reaction through somatic hypermutation that is regulated by Tfh cells and Tfrs, demonstrating the ability of CTLA-4 blockade to regulate the germinal center in a manner that could support a better functional antibody response [40,86]. In fact, we found that the increased levels of higher avidity Env-specific IgG were capable of mediating ADCC (Figure 7). ADCC has been the only significant correlate of protection identified in clinical efficacy trials evaluating candidate HIV vaccines [4,8,19,20]. We observed ADCC activity in four out of 10 VLP immunized mice. We found that five out of 10 mice receiving CTLA-4 blockade combined with VLP immunization, exhibited higher HIV-1BaL-specific ADCC titers, likely reflective of the higher Env-specific IgG present in the serum (Figure 7). Notably, using XC-34 cell generated VLPs in mice can lead to antibodies that recognize surface proteins other than HIV-1 envelope proteins as seen with the nonspecific killing of SIVmac251 infected CEM cells, and this cross reactivity is subtracted out in the calculation of HIV-1BaL-specific ADCC activity. The ADCC responses we observed in this study are in concordance with a previously published macaque study that demonstrated that CTLA-4 blocking antibodies could improve ADCC activity when used in combination with a melanoma vaccine [22]. Our study shows that CTLA-4 blockade can increase germinal center responses, as indicated by increased antigen-specific Tfh activity and increased and accelerated polyclonal B-cell activation resulting in an antibody response with higher avidity and ADCC. Our study was able to detect subtle, but statistically significant increases in avidity, which resulted in the detection of some ADCC activity, although this failed to reach statistical significance. To truly understand the significance of the increased antibody responses, studies in macaques or humanized mouse models would need to be performed.

An interesting and novel finding in our study was that CTLA-4 blockade, during HIV VLP immunization, could accelerate the immune response induction, indicated by the early initiation of T-dependent class switching to HIV-specific IgG2c, elevated APRIL levels after a single immunization, and the presence of high levels of ADCC activity after 2 VLP immunizations (Figure 4C,D, Figure 5A and Figure 6A). APRIL has been shown to modulate antibody class switching, specifically through its receptor, TACI, which promotes T-Independent class switching. This could explain the coinciding upregulation of IgG2c and APRIL after a single VLP immunization with CTLA-4 blockade [23,26,87]. Both APRIL and CTLA-4 have been independently linked to the regulation of the germinal center response through the cytokine, IL-10, however, the relationship between IL-10, CTLA-4 blockade, and APRIL in accelerating immune responses has not been investigated [17,26]. The ability of CTLA-4 blockade to accelerate immune responses is an attractive attribute for any vaccine strategy, as this could have utility in settings where vaccines are limited, or in populations in which adherence to follow-up visits for booster immunizations could be a challenge.

Four previous studies have assessed the effect of CTLA-4 blockade on SIV vaccine responses in the SIV/macaque model of HIV and have yielded conflicting results [53,88,89,90]. Three of the four studies evaluated CTLA-4 blockade in a therapeutic vaccination strategy once SIV infection was established [53,88,90]. Two of these studies reported that CTLA-4 blockade during SIV vaccination after established infection resulted in higher viral loads, decreased responsiveness to anti-retroviral therapy, or increased anti-retroviral drug toxicity; however, both of these studies used modified vaccinia Ankara-based SIV vaccines [53,90]. Another study, that established SIV infection, and then used a canary-pox vector-based SIV vaccine strategy similar to that used in the RV144 trial, found that adding CTLA-4 blockade could significantly lower viral loads [88]. One of the studies investigated CTLA-4 blockade in combination with a prophylactic DNA-based SIV vaccine and found an increase in viral load after high-dose intrarectal challenge. This study focused solely on T-cell responses and did not report B-cell or antibody responses [89]. The effect of CTLA-4 blockade during SIV/HIV vaccination is contingent on the type of vaccine that is administered. We used a non-infectious HIV VLP-based vaccine, capable of directly activating B-cells and dendritic cells and determined that CTLA-4 blockade can alter B-cell responses during HIV VLP immunization resulting in increased antigen-specific antibody responses with higher avidity that are capable of mediating ADCC [31,34].

Inducing cellular memory is imperative for any effective vaccine. We used the markers CD44 and CD62L to identify memory T-cells and found a significant increase in CD44Hi CD62LLo memory CD4 T-cells in VLP immunized mice, in the presence or absence of CTLA-4 blockade, which has been shown in previous VLP studies (Figure 8A) [31]. We used the AID-Cre mouse model to evaluate germinal center activation through irreversible fluorescent labeling of the enzyme, AID, which is activated in B-cells undergoing the germinal center reaction [38]. We assessed B-cell memory using CD73 on bone marrow cells isolated 10 weeks after the final immunization and found a significant increase in AID-activated memory B-cells with VLP immunization alone (Figure 8B). VLPs are capable of directly binding and activating B-cells. In this study, we demonstrate that VLPs can also induce B-cell memory, which make them attractive vaccine candidates for targeting B-cell responses [34]. Altogether, our results demonstrate that VLPs alone are sufficient to induce memory T-cell and B-cell responses and that addition of CTLA-4 blockade does not significantly affect cellular memory.

We investigated how CTLA-4 blockade, in the setting of a clinically relevant HIV VLP-based vaccine, can influence B-cell responses. In concordance with what has been previously shown in the literature, we have demonstrated that CTLA-4 blockade can increase antigen-specific antibody responses [16,17,18]. Our study expands upon the known effects of CTLA-4 blockade regulating the germinal center responses by interrogating the functional qualities of the vaccine-elicited antibodies that are generated. Several studies have shown that deletion of CTLA-4 can result in the expansion of Tfh cells [12,13,16]. We have demonstrated that a vaccine strategy utilizing CTLA-4 blocking antibodies can specifically expand IL-21 secreting antigen-specific Tfh cells (Figure 2A). We have also found that the elevated levels of antibodies have a higher avidity and can result in higher ADCC titers (Figure 6; Figure 7). Some limitations of our study are that we did not include an immunization group that received VLP + isotype control or a group receiving only anti-CTLA-4 Ab in all of our analyses. We performed ELISA analysis on sera from mice treated with only anti-CTLA-4 Ab without any VLPs and did not observe any antigen-specific antibody responses (data not shown). Although these additional control groups would be ideal, we do not expect that they would result in any significant changes in our study’s conclusions.

## 5. Conclusions

Our study showed that CTLA-4 blockade in combination with HIV VLP vaccine results in increased CD4+ T-cell activation with increased IL-21+Env-specific Tfh cells, and significantly increased HIV Gag- and Env-specific IgG which resulted in higher-avidity antibodies capable of mediating ADCC. Using CTLA-4 blockade only without vaccines in C57BL/6J mice seems to have no effect on the CD4+ and CD8+ T-cell activation in mice under the current experimental conditions. Furthermore, addition of CTLA-4 blockade in HIV VLP vaccination can accelerate T-cell dependent class switching and induce significantly high serum levels of the B-cell survival factor APRIL, after a single immunization. Increased and accelerated levels of high-avidity class-switched Env- and Gag-specific IgG1 and IgG2c subtypes are indicative of increased polyclonal B-cell activation, which demonstrate ADCC activity that could contribute to protection. Our study reveals that combining HIV VLPs with CTLA-4 blockade increases antigen-specific B-cell responses, in a novel strategy, which could show utility in future vaccine strategies.

## Figures and Tables

**Table 1:** Neutralizing antibody analysis. Serum samples collected at sacrifice from Cohorts 1 and 2 (*n* = 30) were analyzed for neutralizing antibody activity using TZM-bl assay against the control virus SVA-MLV, HIV-BaL.26, and HIV MN.3. Values reported above are the serum dilution at which relative luminescence units (RLUs) were reduced 50% as compared with virus control wells (no serum). Titers against HIV are considered positive for neutralizing antibody activity based on the criterion of signal >3x that against the negative control virus and >3x the signal of animals in the vaccine control group. No statistical differences were found between groups.

Specimen ID	Specimen Collection Time	SVA-MLV Negative Control	HIV-MN.3 Clade B Tier 1a	HIV-BaL Clade B Tier 1b
PBS 1	10 days post-boost 1	59	180	49
PBS 2		<20	<20	<20
PBS 3		48	134	44
PBS 4		72	220	39
PBS 5		22	22	30
PBS 6	7 days post-boost 2	220	302	474
PBS 7		25	<20	37
PBS 8		<20	55	23
PBS 9		<20	25	<20
PBS 10		<20	55	21
VLP 1	10 days post-boost 1	22	185	57
VLP 2		<20	217	21
VLP 3		<20	66	34
VLP 4		<20	129	26
VLP 5		27	104	39
VLP 6	7 days post-boost 2	<20	233	<20
VLP 7		<20	121	34
VLP 8		<20	198	<20
VLP 9		65	255	78
VLP 10		24	137	44
VLP + anti-CTLA-4 Ab 1	10 days post-boost 1	<20	55	25
VLP + anti-CTLA-4 Ab 2		<20	<20	22
VLP + anti-CTLA-4 Ab 3		<20	77	35
VLP + anti-CTLA-4 Ab 4		22	243	27
VLP + anti-CTLA-4 Ab 5		77	148	90
VLP + anti-CTLA-4 Ab 6	7 days post-boost 2	<20	<20	<20
VLP + anti-CTLA-4 Ab 7		<20	<20	<20
VLP + anti-CTLA-4 Ab 8		<20	203	<20
VLP + anti-CTLA-4 Ab 9		<20	248	<20
VLP + anti-CTLA-4 Ab 10		<20	284	<20

## Supplementary Materials

The following are available online at https://www.mdpi.com/2076-393X/8/2/284/s1, Figure S1: Immunization Strategy, Figure S2: CTLA-4 blockade enhances CD4 T-cell activation, Figure S3: CTLA-4 blockade alone do not enhance baseline CD4 T-cell activation, Figure S4: CTLA-4 blockade enhances Env-specific IL-21 secreting Tfh, Figure S5: VLP Immunization increases germinal center B-cells, Figure S6: Original APRIL Western blot: Serum from cohort 2 was diluted 1:100 in RIPA and Laemmli buffer and loaded for SDS-PAGE, Figure S7: Memory T-cell and B-cell responses are CTLA-4 blockade independent, Figure S8: VLPs express Env in a conformation recognizable by broadly neutralizing antibodies, Table S1: CTLA-4 blockade amplifies serum HIV Env-specific antibody responses, Table S2: CTLA-4 blockade amplifies serum HIV Gag-specific antibody responses.
