# Supplementary material for: CTLA-4 Blockade, during HIV Virus-Like Particles Immunization, Alters HIV-Specific B-Cell Responses
Source: Vaccines (Basel). 2020 Jun 6;8(2):284. doi: 10.3390/vaccines8020284 (PMC7349993; doi:10.3390/vaccines8020284)
Supplement: Supplementary file 1 [file vaccines-08-00284-s001.pdf]

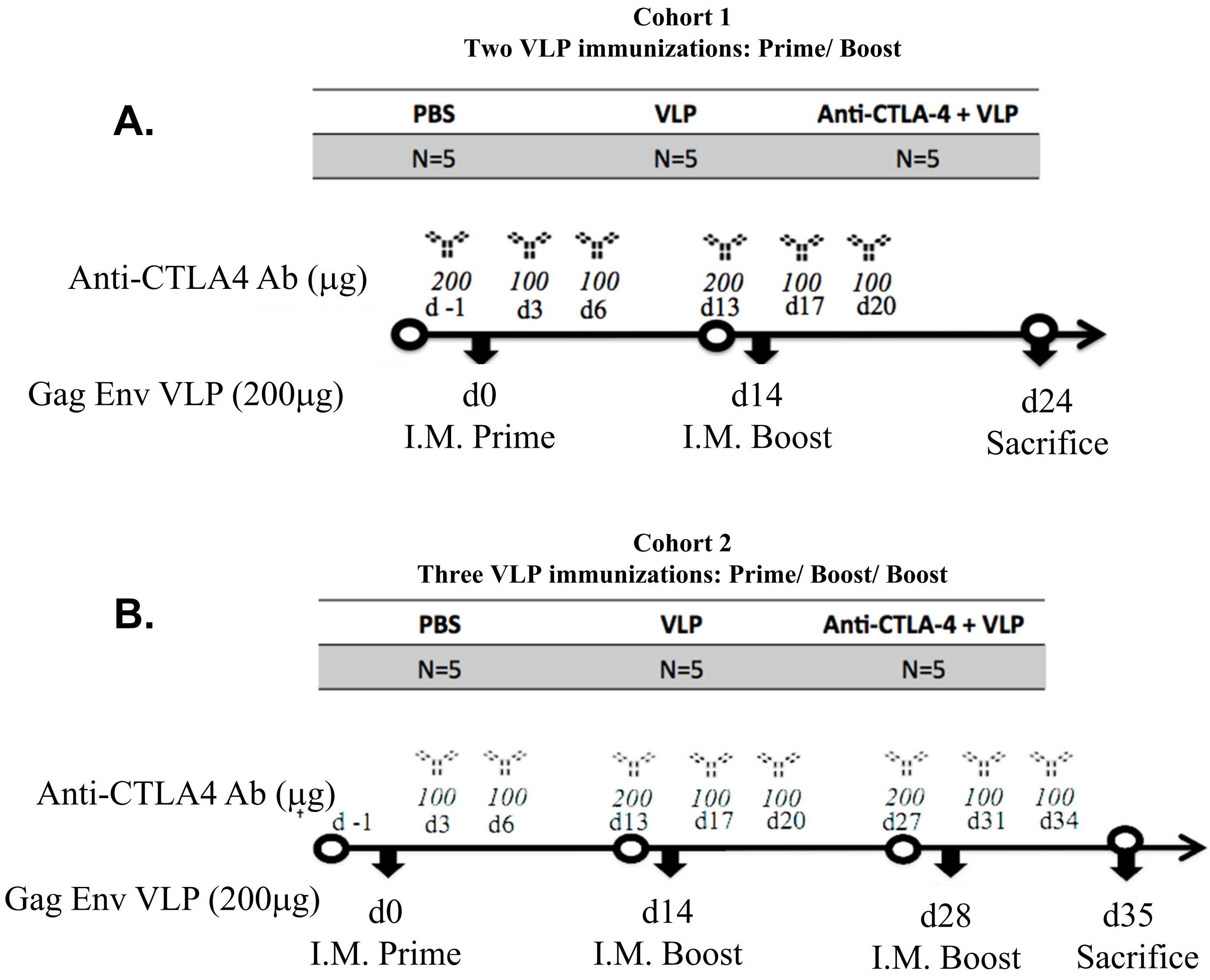

Supplementary Figure S1. Immunization Strategy. Thirty female C57BL/6J Mice were used in two immunization studies. In cohort 1 (**A**), mice were immunized two times (i.m.) with 200 µg of VLPs expressing HIV Gag IIIB and $Envelope_{BaL}$ into the quadriceps. Mice received 200 µg of anti-CTLA-4 blocking antibody one day before each immunization and two additional 100 µg (i.p) doses 3 and 6 days after VLP immunization as described above. In cohort 2, mice received an additional VLP immunization + CTLA-4 blockade in the same manner as study 1, for a total of three intra-muscular VLP immunizations and 9 injections of anti-CTLA-4 (**B**). Prior to injection of anti-CTLA-4 one day before each VLP immunization, blood was drawn through sub-mandibular bleed and vaginal wash was collected in a volume of 100 µL of PBS with protease inhibitor and collection of these specimens are indicated by an open circle on the immunization timeline.

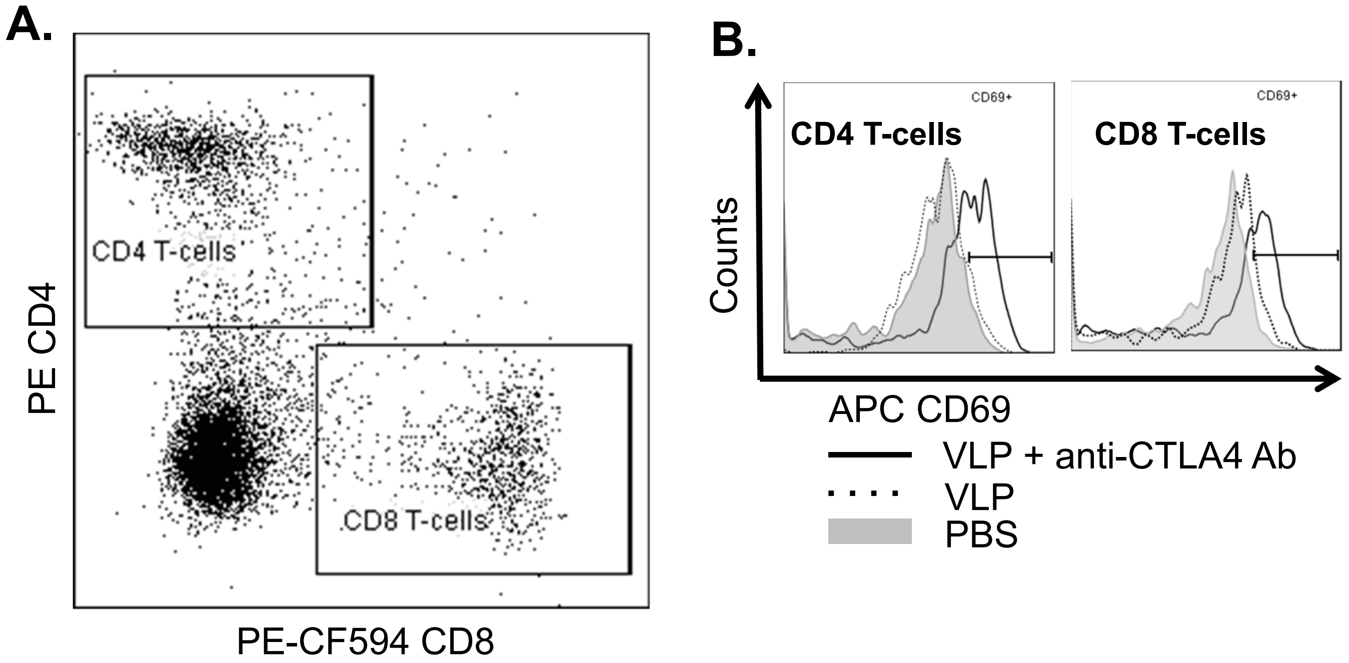

Supplementary Figure S2. CTLA-4 blockade enhances CD4 T-cell activation. Splenocytes from cohort 1 were harvested 10 days after receiving their second VLP dose, and 4 days after receiving their final injection of anti-CTLA-4 Ab. **(A)** $CD3^+$ T-cells were pre-gated and CD4 and CD8 discrimination gating was performed. **(B)** A representative animal from each immunization group shows CD69 expression on the $CD4^+$ T-cell compartment and $CD8^+$ T-cell compartment.

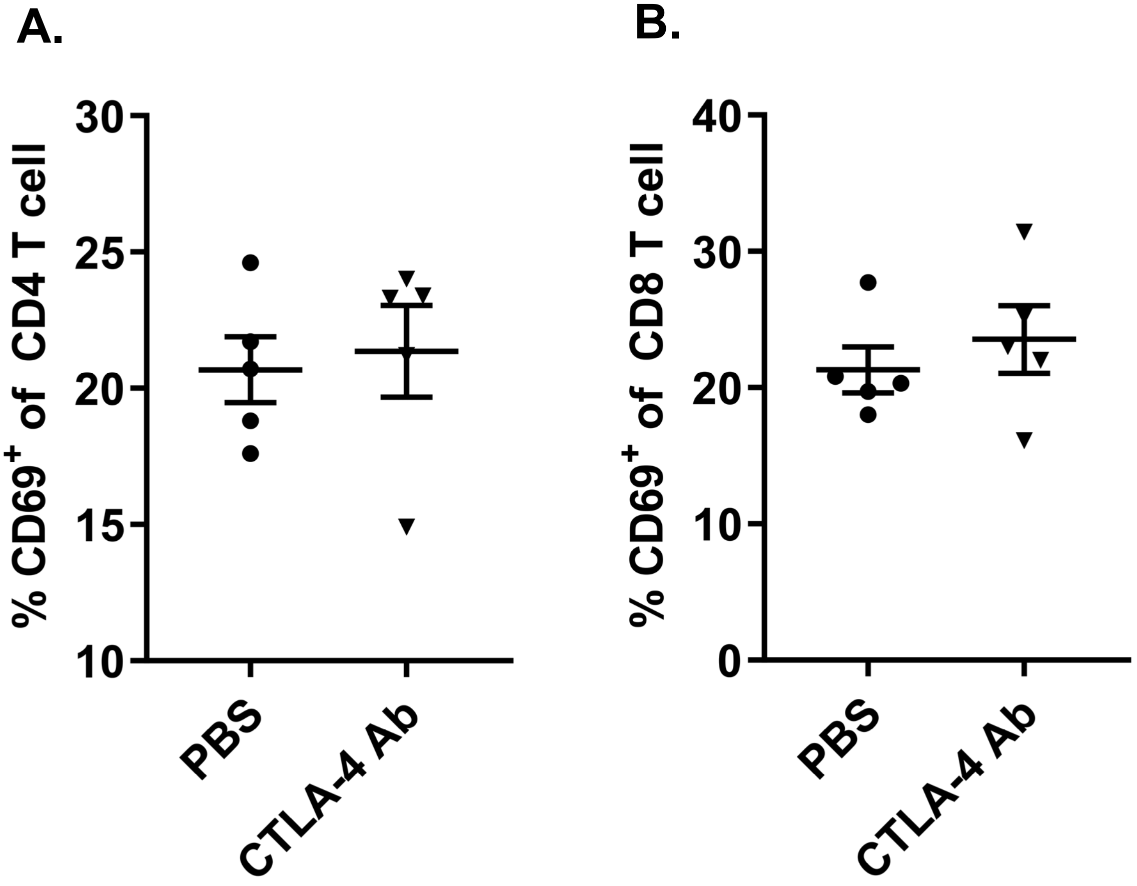

Supplementary Figure S3. Supplementary Figure S2. CTLA-4 blockade alone do not enhance baseline CD4 T-cell activation. Splenocytes were harvested and compared between PBS and anti-CTLA-4 Ab treated group. CD3+ T-cells were pre-gated and CD4 and CD8 discrimination gating was performed. Cumulative results from all animals are shown for (**A**) CD4+CD69+T-cells and (**B**) CD8+CD69+T-cells. Statistical significance was determined using a one-way ANOVA and Tukey post-hoc analysis for multiple comparisons.

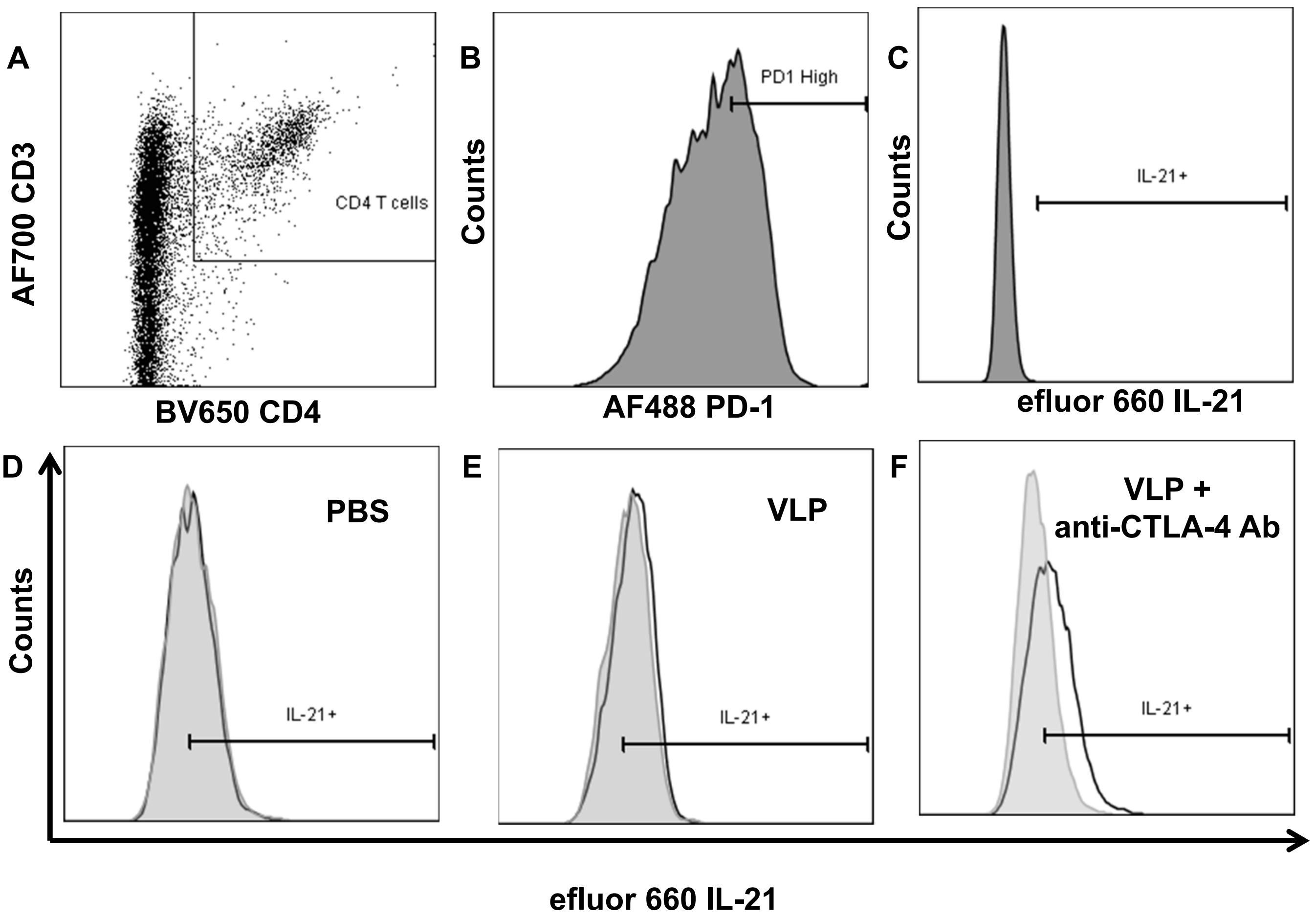

Supplementary Figure S4. CTLA-4 blockade enhances Env-Specific IL-21 secreting Tfh. Lymph Nodes were harvested from cohort 2 at sacrifice, 7 days after they received their last VLP immunization and the antigen-specific follicular T-cell assay was performed. Cells were analyzed on LSR-Fortessa and FlowJo software was used for data analysis. **(A)** $CD4^+$ T-cells were identified as CD3 $AF700^+$ CD4 $BV650^+$. **(B)** $CD4^+$ T-cells with high levels of PD-1 were gated as shown in. **(C)** IL-21 Positivity was gated based off unstained LMNCs. A representative animal from each group (**D**) PBS, (**E**) VLP, (**F**) VLP + anti-CTLA-4 blockade, is shown with non-stimulated $IL\text{-}21^+$ population from $PD\text{-}1^{Hi}$ $CD4^+$ T-cells designated as the grey histogram, while the Env-stimulated $IL\text{-}21^+$ population from $PD\text{-}1^{Hi}$ $CD4^+$ T-cells are designated as the histogram outlined in black.

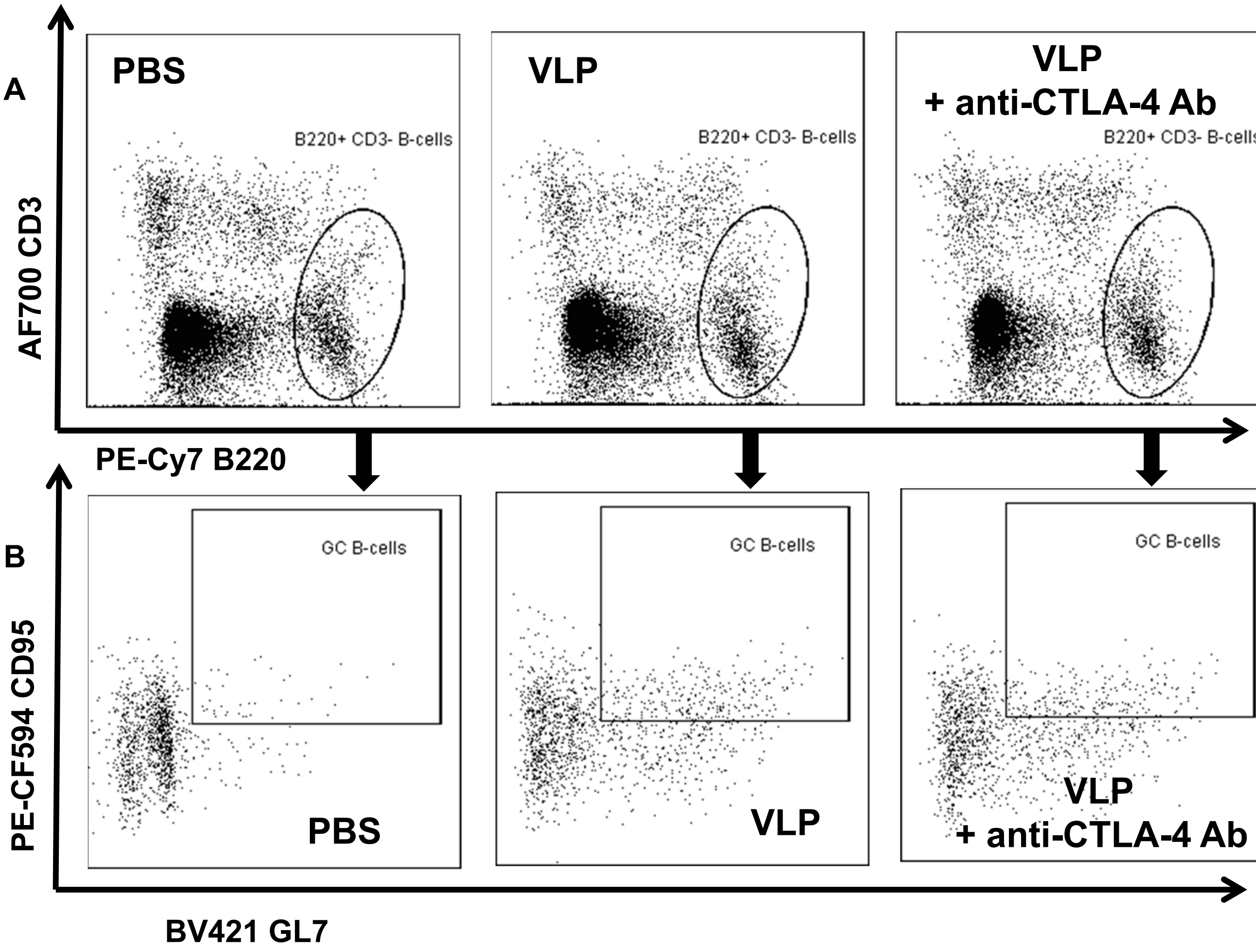

Supplementary Figure S5. VLP Immunization increases germinal center B-cells. LMNCs were isolated from lymph nodes harvested from cohort 2 mice, 7 days after their third and final VLP immunization. LMNCs were stained for CD3 AF700, B220 PE-Cy7, CD95 PE-CF594, and GL7 BV421. **(A)** Gating Strategy: B-cells were gated as $CD3^-$, $B220^+$, **(B)** and germinal center B-cells were identified as the percentage of those cells $CD95^+$ $GL7^+$.

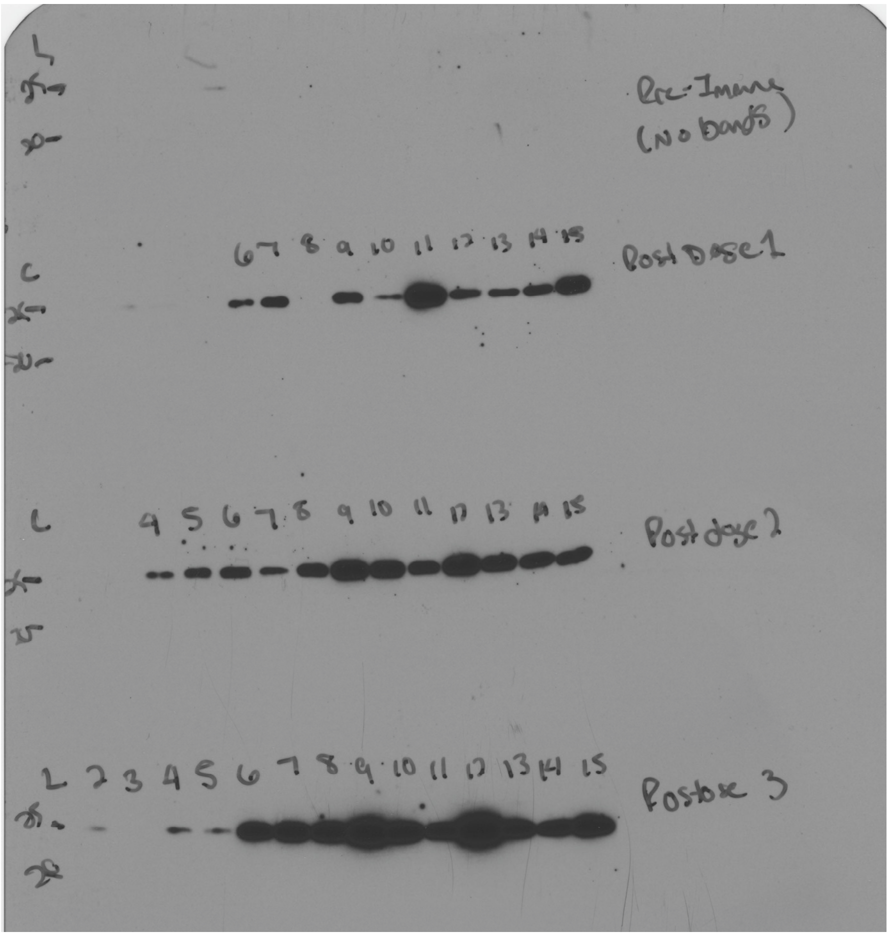

Supplementary Figure S6. Original APRIL Western blot. Serum from cohort 2 was diluted 1:100 in RIPA and Laemmli buffer and loaded for SDS-PAGE. Each 15-well gel was loaded with ladder and serum from 4 PBS mice, 5 VLP immunized mice, and 5 immunized VLP + anti-CTLA-4 Ab mice.

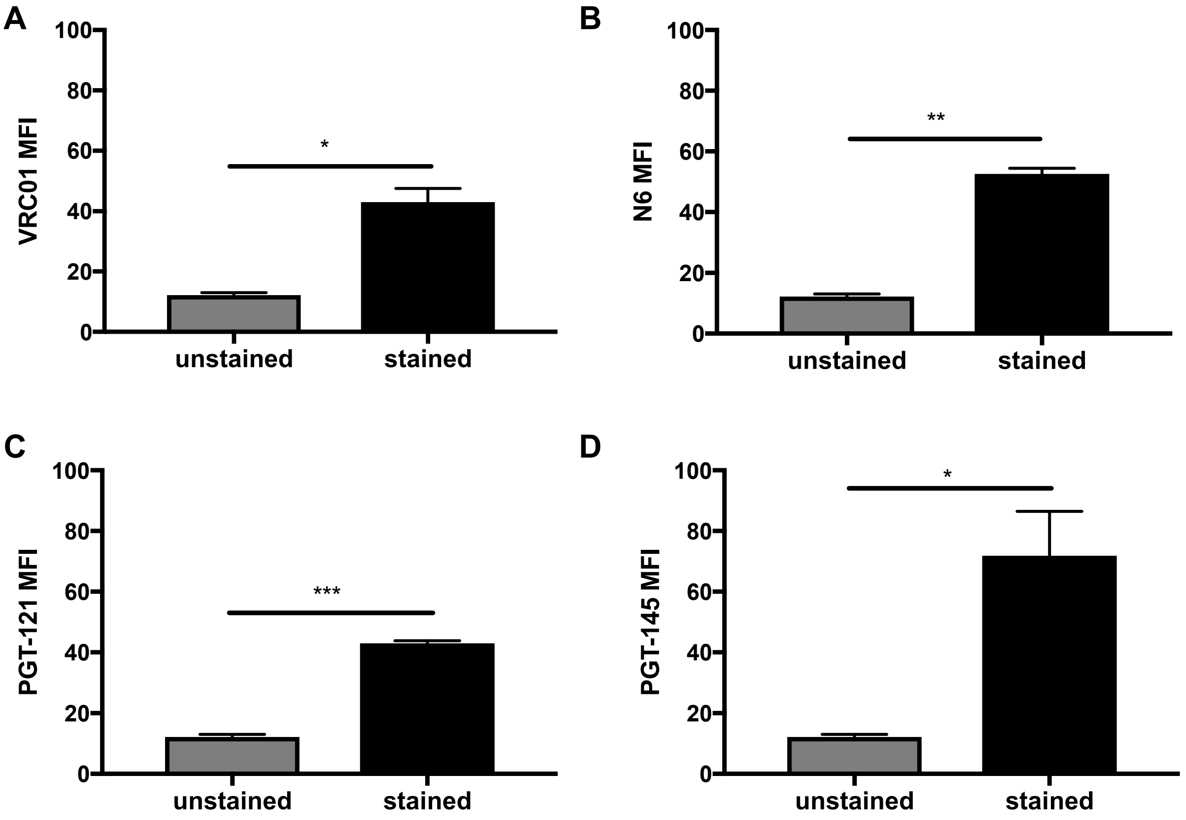

Supplementary Figure S7. VLPs express Env in a conformation recognizable by broadly neutralizing antibodies. XC-34 cells, from which VLPs are produced, were stained with anti-Env broadly neutralizing antibodies (2 μg/mL) followed by staining with anti-human IgG-AF488 secondary antibody (stained), or treated with anti-human IgG-AF488 secondary antibody only (unstained). Graph represents MFI of two independent staining experiments of XC-34 cells . Significance was determined using an unpaired T-Test. ***$p < 0.001$, **$p < 0.01$ *$p < 0.05$.

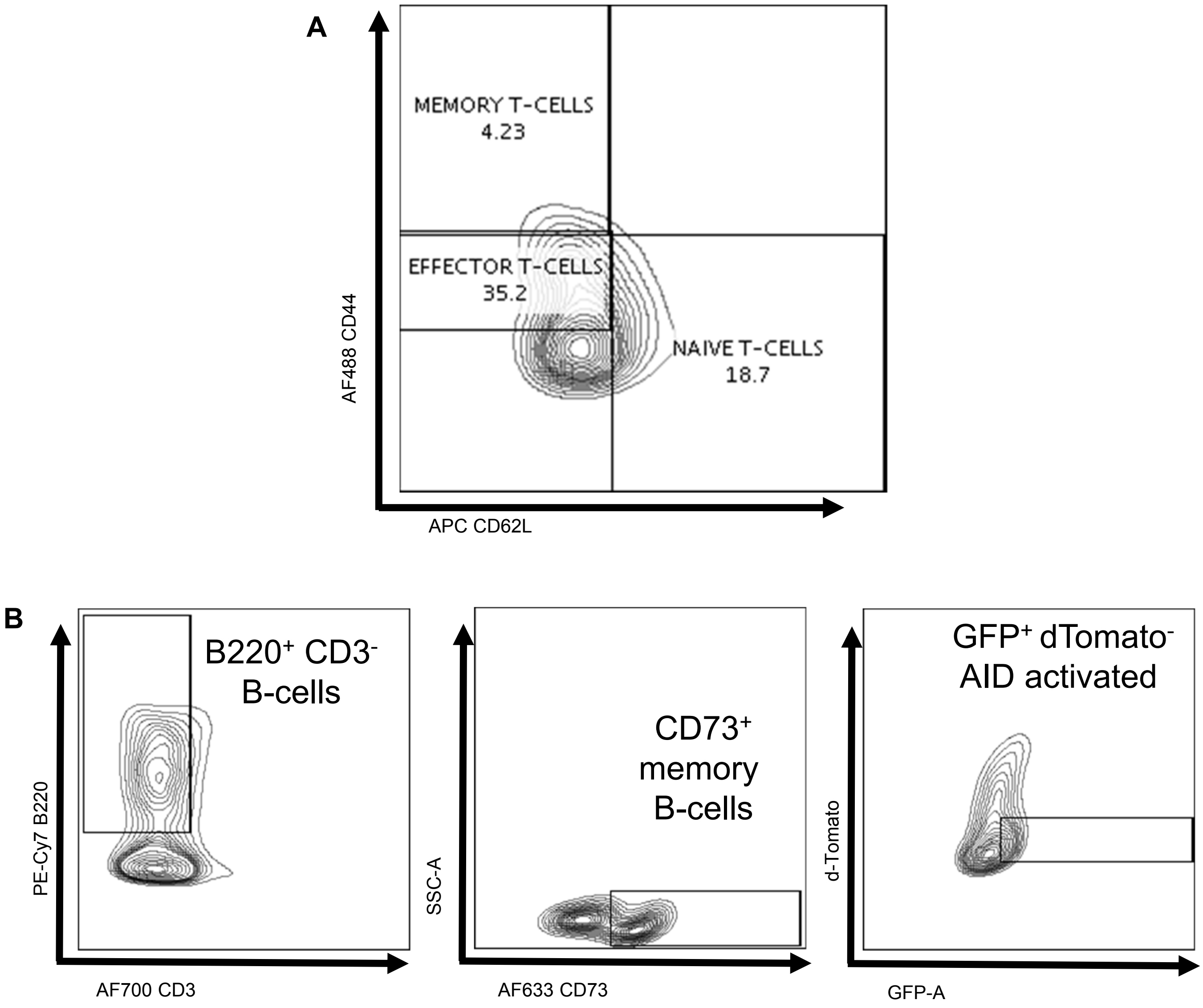

Supplementary Figure S8. Memory T-cell and B-cell responses are CTLA-4 blockade independent. Splenocytes from cohort 1 were stained with CD3 AF700, CD4 BV650, CD8 BV786, CD62L APC, and CD44 FITC. **(A)** Memory T-cells were designated as CD4 T-cells that were $CD44^{hi}$ $CD62L^{Lo}$. **(B)** AID memory B-cells were harvested from the bone marrow of AID-reporter mice (n=11) 10 weeks after the third VLP immunization and stained with CD3 AF700, B220 PE-Cy7, and CD73 AF650. Gating strategy to identify GFP^{+} $dTomato^{-}$(AID-activation) within the $CD73^{+}$ (memory) B-cell compartment is shown.

	Immunization Group	**Post-Prime** n=10/group Mean (SD)	**Post-Boost 1** n=10/group Mean (SD)	**Post-Boost 2** n=5/group Mean (SD)
**anti-Env IgG1 (µg/mL)**	PBS	<0.01 (0)	<0.01 (0)	0.25 (0.56)
	VLP	<0.01 (0)	10.77 (9.49)	15.85 (12.89)
	VLP +anti-CTLA-4 Ab	1.43 (3.29)	52.71 (42.40)	79.23 (16.47)
**anti-Env IgG2b (µg/mL)**	PBS	<0.01 (0)	<0.01 (0)	<0.01 (0)
	VLP	4.51 (4.68)	3.94 (4.38)	16.73 (10.20)
	VLP +anti-CTLA-4 Ab	11.27 (8.59)	6.46 (2.78)	20.54 (10.77)
**anti-Env IgG2c (µg/mL)**	PBS	<0.01 (0)	1.04 (1.67)	<0.01 (0)
	VLP	2.25 (1.67)	53.45 (26.82)	84.05 (36.83)
	VLP +anti-CTLA-4 Ab	5.64 (4.30)	89.24 (90.26)	94.09 (15.78)
**anti-Env IgG3 (µg/mL)**	PBS	<0.01 (0)	0.29 (0.46)	<0.01 (0)
	VLP	0.06 (0.18)	1.24 (1.29)	1.53 (1.97)
	VLP +anti-CTLA-4 Ab	1.14 (1.52)	1.04 (0.74)	0.73 (1.03)

Supplementary Table S1. CTLA-4 blockade amplifies serum HIV Env-specific antibody responses. Post-prime serum was collected 13 days after the first VLP immunization; Post- Boost 1 serum was collected 11-13 days after the second VLP immunization. Post-Prime and Post-Boost 1 time points analyzed serum from 10 mice per immunization group from cohorts 1 and cohorts 2. The Post-Boost 2 time point analyzed serum from the 15 mice (5 per group) in cohort 2 and was collected 7 days after the third VLP immunization. Quantitative ELISAs with standard curves for IgG1, IgG2b, IgG2c, and IgG3 were performed in duplicate on all serum samples from cohorts 1 and 2. The results above are the cumulative results of two independent experiments. The numbers in the table indicate the mean concentration (µg/mL) of Env-specific IgG (Standard Deviation) for each immunization group over time, This data is also represented in histogram form in Figure 4 C,E, and G.

	**Immunization Group**	**Post-Prime** n=10/group Mean (SD)	**Post-Boost 1** n=10/group Mean (SD)	**Post-Boost 2** n=5/group Mean (SD)
**anti-Gag IgG1 (µg/mL)**	PBS	<0.01 (0)	0.20 (0.63)	0.02 (0.04)
	VLP	<0.01 (0)	17.8 (11.68)	28.85 (15.04)
	VLP +anti-CTLA-4 Ab	2.63 (5.97)	111.47 (55.54)	157.27 (61.60)
**anti-Gag IgG2b (µg/mL)**	PBS	0.15 (0.45)	<0.01 (0)	<0.01 (0)
	VLP	1.72 (3.20)	3.16 (3.14)	22.26 (17.14)
	VLP +anti-CTLA-4 Ab	21.60 (19.4)	7.77 (3.19)	25.67 (11.97)
**anti-Gag IgG2c (µg/mL)**	PBS	0.07 (0.23)	0.24 (0.60)	<0.01 (0)
	VLP	0.86 (1.60)	38.30 (28.50)	12.48 (11.11)
	VLP +anti-CTLA-4 Ab	10.80 (9.71)	114.43 (44.05)	85.86 (24.63)
**anti-Gag IgG3 (µg/mL)**	PBS	0.15 (0.31)	0.86 (1.10)	<0.01 (0)
	VLP	0.38 (0.67)	1.19 (1.90)	1.20 (1.64)
	VLP +anti-CTLA-4 Ab	0.01 (0.02)	1.13 (1.43)	0.89 (1.33)

Supplementary Table S2. CTLA-4 blockade amplifies serum HIV Gag-specific antibody responses. Post-prime serum was collected 13 days after the first VLP immunization; Post- Boost 1 serum was collected 11-13 days after the second VLP immunization. Post-Prime and Post-Boost 1 time points analyzed serum from 10 mice per immunization group from cohorts 1 and cohorts 2. The Post-Boost 2 time point analyzed serum from the 15 mice (5 per group) in cohort 2 and was collected 7 days after the third VLP immunization. Quantitative ELISAs with standard curves for IgG1, IgG2b, IgG2c, and IgG3 were performed in duplicate on all serum samples from cohorts 1 and 2. The results above are the cumulative results of two independent experiments. The numbers in the table indicate the mean concentration (µg/mL) of Gag-specific IgG (Standard Deviation) for each immunization group over time, This data is also represented in histogram form in Figure 4 D, F, and H.
